# Emerging *Aeromonas* spp. infections in Europe: characterization of human clinical isolates from German patients

**DOI:** 10.3389/fmicb.2024.1498180

**Published:** 2024-12-18

**Authors:** Keike Schwartz, Maria Borowiak, Eckhard Strauch, Carlus Deneke, Martin H. Richter, The German Aeromonas Study Group

**Affiliations:** Department Biological Safety, German Federal Institute for Risk Assessment (BfR), Berlin, Germany

**Keywords:** Germany, *Aeromonas*, whole genome sequencing, virulence gene profile, One Health, climate change, human pathogenic isolates

## Abstract

Bacteria of the genus *Aeromonas* are widely distributed in water bodies around the world. Some *Aeromonas* species have been identified as human pathogens causing intestinal and a variety of extraintestinal infections. In Germany, information on diseases caused by *Aeromonas* is rare, because *Aeromonas* infections are not notifiable in Germany. To address this information gap and gain better insights, a successful collaboration with human medical diagnostic laboratories within Germany was established and several *Aeromonas* isolates from diseased patients were sent to the *Aeromonas* laboratory of the German Federal Institute for Risk Assessment. 52 clinical *Aeromonas* isolates, of which anonymized patients’ data were available, were selected for further characterization by MALDI-TOF MS, biochemical testing, and whole genome sequencing (WGS). Nearly half of the isolates were from patients older than 60 years, whereas only four isolates were from patients aged up to 10 years. 30 isolates originated from stools of patients with diarrhea/(gastro-)enteritis and 22 strains were from patients with diverse extraintestinal infections, such as wound infection, septicemia, bursitis, abscesses, cholangitis, urinary tract infections, and pneumonia. Taxonomical identification revealed following predominant species: *A. veronii* biovar *sobria* (18 isolates), *A. caviae* (17 isolates), and *A. hydrophila* (nine isolates). Two *A. salmonicida* isolates and one isolate each of *A. dhakensis, A. bestiarum,* and *A. encheleia* were also identified. Three closely related intestinal isolates could not be assigned to a recognized *Aeromonas* species. The clustering of strains based on virulence factor profile resulted in a grouping that closely resembles the clustering of the phylogenetic tree suggesting that the profiles are specific for each species. Our study on clinical *Aeromonas* isolates characterizes for the first time human pathogenic strains isolated in Germany. These bacteria are important microorganisms within the One Health context because of their ubiquitous presence in the environment and as a cause of opportunistic infections in humans and animals. Infections caused by *Aeromonas* bacteria show a seasonality with increased infection rates in warmer periods. In view of climate change, *Aeromonas* bacteria are regarded as emerging pathogens and research is required to determine the reservoirs in One Health sources from which human infections may arise.

## 1 Introduction

*Aeromonas* bacteria are distributed in aquatic environments throughout the world. They can be isolated from rivers, lakes, ponds, seawater (estuaries), drinking water, groundwater, wastewater, and sewage in various stages of treatment (Janda and Abbott, 2010). In addition, *Aeromonas* bacteria are also found in soil and on plants (Lamy et al., 2022) and were detected in insects such as chironomids (Laviad and Halpern, 2016) as well as in the intestinal tract of crustaceans (Zhao et al., 2018), fish (Ofek et al., 2021), birds (Laviad-Shitrit et al., 2018), and mammals (Lamy et al., 2022).

Whereas *Aeromonas* bacteria have been known as fish pathogens for decades, it was recognized later that some *Aeromonas* species can cause serious illness, sometimes fatal, in humans (Janda and Abbott, 2010). More than 30 *Aeromonas* species have been described and the most frequently isolated strains from human clinical samples belong to the species *A. hydrophila*, *A. caviae*, and *A. veronii* biovar *sobria* (Pessoa et al., 2022). Additionally, strains from other *Aeromonas* species have been found in human specimen, but their occurrence can vary depending on the countries (Teodoro et al., 2022). In the past 20 years, human infections have been reported not only in Asian countries, but increasingly also in Europe. Among them were numerous gastrointestinal infections and locally acquired extraintestinal infections, including wound and skin infections, soft tissue infections, pneumonias, and septicemias. Many infections were associated with previous water contact during bathing or fishing or contact with animal foods (Avolio et al., 2009; Spadaro et al., 2014; Couturier et al., 2017; Fouquet et al., 2018; Ganiatsa et al., 2020). In addition, *Aeromonas*-associated cases of hemolytic uremic syndrome (HUS) have been reported (Figueras et al., 2007; Castellano-Martinez et al., 2019), in which foodborne transmission was assumed. Case reports from Switzerland, the United Kingdom (UK), Belgium, the Netherlands, and Finland highlight the increasing importance of *Aeromonas* infections also in Central Europe and the North Sea and Baltic Sea regions (Elwitigala et al., 2005; Adamski et al., 2006; Borger van der Burg et al., 2006; Bossi-Küpfer et al., 2007; Rutteman et al., 2017; Bosch et al., 2019). Although gastroenteritis, wound infections, and septicemia are frequently observed in immunocompetent patients, the immunocompromised and elderly appear to have an increased risk of developing disease. Fatal courses of disease are particularly common in septicemic conditions, which can develop secondarily from both wound infection and gastroenteritis (Pund and Theegarten, 2008; Nikiforov et al., 2014; Fernández-Bravo and Figueras, 2020).

Whereas human *Aeromonas* infections following natural disasters in the Asian region were often clearly attributable to contact with contaminated water and/or contaminated soil (Pund and Theegarten, 2008), the significance of sources or reservoirs with respect to human infections in Germany remains largely unclear to date. This could be because *Aeromonas* spp. infections are not notifiable and hence not subject to mandatory reporting in Germany. Therefore, no reliable data on incidence currently exist. Against the background of the first food- and waterborne outbreaks in Europe [European Food Safety Authority (EFSA) and European Centre for Disease Prevention and Control (ECDC), 2023] and some reports of cases in Germany, the Federal Institute for Risk Assessment (BfR) started to collect bacteria of the *Aeromonas* group that were isolated from diseased patients in Germany.

In the course of this study, successful collaborations with a number of human medical diagnostic laboratories within Germany were established and several *Aeromonas* isolates from clinical sources were sent to the German Federal Institute for Risk Assessment. Information on diseased patients was anonymized. However, several disease-associated *Aeromonas* spp. isolates have been preserved, originating from patients with intestinal and extraintestinal infections.

The aim of our study was to characterize German clinical isolates and to obtain data on the relevant traits of human pathogenic strains that can be used to search for their sources and/or reservoirs in the environment, in food, and in animals according to the One Health concept. For this purpose, the clinical isolates were analyzed using whole genome sequencing. Furthermore, matrix-assisted laser desorption/ionization time-of-flight mass spectrometry (MALDI-TOF MS) and biochemical characterization were applied for diagnostics. In this study, 52 isolates from intestinal and extraintestinal samples were selected and investigated.

## 2 Materials and methods

### 2.1 Bacterial isolates and cultivation

A total of 52 *Aeromonas* spp. isolates from German patients (*n* = 51) with intestinal or extraintestinal infections were investigated in this study. The isolates were collected between 2016 and 2019 and were obtained from different human medical diagnostic laboratories and medical care centers of the German federal states Brandenburg, Thuringia, North Rhine-Westphalia, and Mecklenburg-Western Pomerania, a coastal state bordering the Baltic Sea.

Pure cultures of *Aeromonas* spp. isolates were routinely stored at −80°C in tryptic soy broth with 40% (*v*/*v*) sterile glycerol. Prior to downstream analysis, bacteria were plated onto tryptic soy agar (Merck, Darmstadt, Germany) and incubated overnight at 37°C.

### 2.2 Mass spectrometric analysis

Genus confirmation of the clinical *Aeromonas* spp. isolates was performed by whole-cell MALDI-TOF MS analysis.

For sample preparation, the direct transfer procedure was employed according to the manufacturer’s protocol (Bruker Daltonik, Bremen, Germany). Cell material from a single colony of an overnight culture was applied to a stainless steel MSP 96 target (Bruker Daltonik) using a sterile toothpick.

MALDI-TOF MS measurements were conducted on a Bruker Microflex LT/SH benchtop mass spectrometer. Spectra data were automatically acquired by using the AutoXecute method (MBT_AutoX) in flexControl v3.4 (Bruker Daltonik). Analysis of captured mass spectra was performed using automated control and the MBT Compass software v4.1.100 and reference library v11.0 (Bruker Daltonik).

### 2.3 Whole genome sequencing and bioinformatic analyses

To gain insights into the genotypic traits of German clinical *Aeromonas* spp. isolates, the incoming isolates were successively subjected to whole genome sequencing (WGS) over the years. After overnight cultivation in tryptic soy broth at 37°C, genomic DNA of the isolates was extracted using the PureLink Genomic DNA Mini Kit (Thermo Fisher Scientific, Waltham, MA, United States). Details on DNA library preparation and DNA sequencing using Illumina technologies (Illumina, San Diego, CA, United States) are given in Supplementary Table S1.

Initial analysis of WGS data was performed with the bioinformatics pipeline AQUAMIS v1.3.8 (Deneke et al., 2021) that implemented fastp v0.20.1 (Chen et al., 2018) for trimming and quality control of sequencing reads, shovill v1.1.0 (Seemann, 2020) [based on SPAdes v3.14.1 (Prjibelski et al., 2020)] for *de novo* assembly of trimmed reads, and QUAST v5.0.2 (Gurevich et al., 2013) for assembly quality control.

The genome assemblies obtained from AQUAMIS served as a basis for species determination. Species pre-identification was performed using the Bacterial and Viral Bioinformatics Resource Center’s (BV-BRC) Similar Genome Finder (SGF) tool (https://www.bv-brc.org/app/GenomeDistance; v3.28.9). In SGF analyses, genome sequences of the *Aeromonas* spp. isolates from German patients as well as all publicly available bacterial and archaeal genomes from the BV-BRC database were included. Starting with the first (best) BV-BRC SGF-match, up to five SGF-based species results were verified using the EzBioCloud’s Average Nucleotide Identity (ANI) calculator tool.^1^ In ANI analyses, genome sequences of the *Aeromonas* spp. isolates from German patients and *Aeromonas* spp. type strain genomes (reference genomes) from the National Center for Biotechnology Information’s (NCBI) Reference Sequence (RefSeq) database served as input files. Details are given in the “Accession numbers” section. *Aeromonas* isolates that could not be reliably identified at the species level (OrthoANIu values ≤95%; Yoon et al., 2017) were referred to as “*Aeromonas* sp.”

To analyze the phylogenetic relationship between the 52 clinical *Aeromonas* isolates and seven NCBI reference strains representing the species found in this study, a phylogenetic study was performed including three *Tolumonas* reference strains as outgroup. First, the core genome shared by all 62 strains was identified based on the provided assemblies using Panaroo v1.3.4 (Tonkin-Hill et al., 2020). The resulting filtered core genome alignment produced by Panaroo was further used as input to create a maximum-likelihood-based phylogenetic tree using IQ-TREE v2.2.0 (Nguyen et al., 2015).

For a more detailed phylogenetic investigation of the main *Aeromonas* species detected in this study (*A. caviae*, *A. hydrophila*, and *A. veronii*), three SNP analysis studies were conducted using trimmed Illumina data as input and the respective NCBI reference genomes as reference. SNP calling was performed using the snippySnake pipeline v1.3.0 (Lüth et al., 2021) that utilizes snippy v4.6.0^2^ for variant calling. The core SNPs were used to generate maximum-likelihood-based phylogenetic trees using IQ-TREE v2.2.0 (Nguyen et al., 2015).

For phylogenetic trees utilizing IQ-TREE, the General Time Reversible (GTR) model was used. Branch support values [Shimodaira-Hasegawa-like approximate likelihood ratio (SH-aLRT)] were calculated with 1,000 replicates. The phylogenetic trees were visualized with iTOL v6.

The number of SNP differences between each pair of *A. caviae* isolates, *A. hydrophila* isolates, and *A. veronii* isolates was calculated by using snp-dists v0.8.2^3^ within the snippySnake pipeline.

Presence/absence analysis of genetic virulence determinants was performed using the Bacterial Characterization (BakCharak) pipeline (https://gitlab.com/bfr_bioinformatics/bakcharak; v3.0.4) that implemented the ABRicate tool (https://github.com/tseemann/abricate; v1.0.1) with a manually curated database of the virulence factor database (VFDB).^4^ The database was derived from the VFDB DNA full dataset (setB) and only included *Aeromonas*-specific virulence genes. Functional categories and subcategories of the 288 genes analyzed in this study are given in Table 1. In BakCharak analysis, the genome assemblies of the clinical isolates served as input files. For *in silico* predictions, a gene identity level of ≥80% and a gene coverage level of ≥50% was used.

**Table 1:** The functional category and subcategory of 288 *Aeromonas* virulence genes analyzed in this study.

Functional category and number of genes	Functional subcategory*^a^* and number of genes	Virulence genes
Adherence (*n* = 58)	Flp type IV pili (*n* = 13)	*flp1*, *flpA*, *flpB*, *flpC*, *flpD*, *flpE*, *flpF*, *flpG*, *flpH*, *flpI*, *flpJ*, *flpK*, *flpL*
	MSHA type IV pili (*n* = 17)	*mshA*, *mshB*, *mshC*, *mshD*, *mshE*, *mshF*, *mshG*, *mshI*, *mshI1*, *mshJ*, *mshK*, *mshL*, *mshM*, *mshN*, *mshO*, *mshP*, *mshQ*
	Tap type IV pili (*n* = 23)	*ASA_RS18105*, *tapA*, *tapB*, *tapC*, *tapD*, *tapF*, *tapM*, *tapN*, *tapO*, *tapP*, *tapQ*, *tapT*, *tapU*, *tapV*, *tapW*, *tapY1*, *tapY2*, *tppA*, *tppB*, *tppC*, *tppD*, *tppE*, *tppF*
	Type I pili (*n* = 5)	*fimA*, *fimC*, *fimD*, *fimE*, *fimF*
Effector delivery system (*n* = 86)	Exe T2SS (*n* = 14)	*exeA*, *exeB*, *exeC*, *exeD*, *exeE*, *exeF*, *exeG*, *exeH*, *exeI*, *exeJ*, *exeK*, *exeL*, *exeM*, *exeN*
	T3SS (*n* = 41)	*acr1*, *acr2*, *acrG*, *acrH*, *acrR*, *acrV*, *aopB*, *aopD*, *aopN*, *aopX*, *ascB*, *ascC*, *ascD*, *ascE*, *ascF*, *ascG*, *ascH*, *ascI*, *ascJ*, *ascK*, *ascL*, *ascN*, *ascO*, *ascP*, *ascQ*, *ascR*, *ascS*, *ascT*, *ascU*, *ascV*, *ascX*, *ascY*, *ati1*, *exsA*, *exsB*, *exsC*, *exsD*, *exsE*, *sycH*, *sycO*, *sycX*
	T3SS secreted effectors (*n* = 6)	*ABH06556, aexT*, *aopH*, *aopO, aopP, ati2*
	T6SS (*n* = 24)	*AHA_RS09305*/*ASA_RS12290*, *atsA*, *atsB*, *atsC*, *atsD*, *atsG*, *atsH*, *atsI*, *atsJ*, *atsK*, *atsL*, *atsP*, *atsQ*, *atsS*, *clpB*, *dotU*, *hcp*, *hcp1*, *vasH*, *vasK*, *vgrG2*, *vgrG3*, *vipA*, *vipB*
	T6SS secreted effectors (*n* = 1)	*vgrG1*
Exotoxin (*n* = 15)	Aerolysin (*n* = 4)	*AAC44637*, *AAN77507*, *ABJ52834*, *aerA*
	Extracellular hemolysin AHH1 (*n* = 1)	*ahh1*
	Heat-stable cytotonic enterotoxin, Ast (*n* = 1)	*ast*
	Hemolysin HlyA (*n* = 1)	*hlyA*
	Hemolysin III (*n* = 1)	*AHA_RS17655*/*AHML_RS18555*/*ASA_RS04230*/*B565_RS04085*
	RtxA (*n* = 6)	*rtxA*, *rtxB*, *rtxC*, *rtxD*, *rtxE*, *rtxH*
	Thermostable hemolysin (*n* = 1)	*AHA_RS16280*/*AHML_RS17230*/*ASA_RS05555*/*B565_RS04805*
Nutritional/Metabolic factor (*n* = 10)	Amonabactin (*n* = 1)	*amoA*
	Heme uptake system (*n* = 9)	*ASA_RS16540*, *ASA_RS16545*, *ASA_RS16550*, *ASA_RS16555*, *ASA_RS16570*, *ASA_RS16575*, *ASA_RS16580*, *hutX*, *hutZ*
Motility (*n* = 119)	Polar and/or lateral flagella (*n* = 119)	*AHA_RS06990*/*AHML_RS07540*/*ASA_RS06915*/*B565_RS05765*, *AHA_RS21095*/*ASA_RS00890*, *cheA-2*, *cheB-2*, *cheR-3*, *cheV*, *cheW*, *cheW-2*, *cheY*, *cheZ*, *flaA*, *flaB*, *flaG*, *flaH*, *flaJ*, *flgA*, *flgAL*, *flgB*, *flgBL*, *flgC*, *flgCL*, *flgD*, *flgDL*, *flgE*, *flgEL*, *flgF*, *flgFL*, *flgG*, *flgGL*, *flgH*, *flgHL*, *flgI*, *flgIL*, *flgJ*, *flgJL*, *flgK*, *flgKL*, *flgL*, *flgM*, *flgML*, *flgN*, *flgNL*, *flhA*, *flhAL*, *flhB*, *flhBL*, *flhF*, *flhG*, *fliA*, *fliE*, *fliEL*, *fliF*, *fliFL*, *fliG*, *fliGL*, *fliH*, *fliHL*, *fliI*, *fliIL*, *fliJ*, *fliJL*, *fliK*, *fliL*, *fliM*, *fliML*, *fliN*, *fliNL*, *fliO*, *fliP*, *fliPL*, *fliQ*, *fliQL*, *fliR*, *fliRL*, *flmD*, *flmH*, *flrA*, *flrB*, *flrC*, *lafA*, *lafB*, *lafC, lafE*, *lafF*, *lafK*, *lafS*, *lafT*, *lafU*, *lafX*, *lfgA*, *lfgB*, *lfgF*, *lfgG*, *lfgH*, *lfgK*, *lfgL*, *lfgM*, *lfgN*, *lfhA*, *lfhB*, *lfiE*, *lfiH*, *lfiI*, *lfiJ*, *lfiM*, *lfiN*, *lfiQ*, *lfiR*, *maf-1*, *maf-2*, *maf-5*, *motA*, *motX*, *motY*, *nueA*, *nueB*, *pomA2*, *pomB*, *pomB2*

a Flp, fimbrial low-molecular weight protein; MSHA, mannose-sensitive hemagglutinin; RtxA, repeat in toxin A; Tap, type IV *Aeromonas* pilus; T2SS, type II secretion system; T3SS, type III secretion system; T6SS, type VI secretion system. The superscript letter that introduces the explanation in italics.

An assessment of the occurrence of selected major virulence factors (single genes as well as complex structures like secretion systems) was performed manually under standardized conditions specified in Supplementary Table S2 (see sheet “Assessment Key”). Possible assessment categories for a virulence factor were: probably completely present (c), partially present (p), and absent (a). In general, a virulence factor was considered to be probably completely present if ≥80% of the genes examined in this study were detected. Further, isolates were considered aerolysin-positive if the aerolysin A gene (*aerA*) and/or at least one of the three aerolysin-like hemolysin (ALH) genes tested (*AAC44637*, *AAN77507*, and *ABJ52834*) were present.

For grouping of the isolates based on virulence traits, virulence factor profiles were identified and similarity patterns were determined by Complete linkage using categorical (values) similarity coefficient of ternary data in BioNumerics v7.6.3 (Applied Maths NV, Sint-Martens-Latem, Belgium).

### 2.4 Biochemical characterization

Biovar affiliation of *A. veronii* isolates was determined by biochemical testing using API 20 E strips (bioMérieux, Nürtingen, Germany) according to the manufacturer’s instruction. Isolates that were arginine dihydrolase (ADH)-positive and ornithine decarboxylase (ODC)-negative were assigned to the biovar *sobria* (Martin-Carnahan and Joseph, 2015). *Aeromonas veronii* biovar *veronii* ATCC 35624^T^ (ADH^−^ ODC^+^) and *Aeromonas veronii* biovar *sobria* LMG 3785 (ADH^+^ ODC^−^) were used for quality assurance.

### 2.5 Statistical analyses

The association between patient characteristics and infection prevalence, infection type and *Aeromonas* species, or selected isolate characteristics and present virulence determinants was analyzed by descriptive statistics. To evaluate if observed differences were statistically significant (*p* ≤ 0.05), the two-tailed Fisher’s exact test was applied with 2 × 2 contingency tables in STATISTICA v14.1.0.8 (StatSoft, Hamburg, Germany).

### 2.6 Accession numbers

Raw sequencing reads for the 52 *Aeromonas* spp. isolates from German patients were deposited in the Sequence Read Archive (SRA) at NCBI.^5^ Accession numbers are given in Supplementary Table S1.

For species determination and/or phylogenetic analysis, genome sequences of *Aeromonas* spp. and *Tolumonas* spp. type strains (reference strains) were obtained from the Reference Sequence (RefSeq) database at NCBI.^6^ NCBI RefSeq/assembly accessions are NZ_CDDA00000000.1/GCF_000819745.1 (*Aeromonas bestiarum* CECT 4227^T^), NZ_LS483441.1/GCF_900476005.1 (*Aeromonas caviae* NCTC 12244^T^), NZ_CDBH00000000.1/GCF_000820305.1 (*Aeromonas dhakensis* CIP 107500^T^), NZ_CDDI00000000.1/GCF_000819825.1 (*Aeromonas encheleia* CECT 4342^T^), NC_008570.1/GCF_000014805.1 (*Aeromonas hydrophila* ATCC 7966^T^), NZ_CDBZ00000000.1/GCF_000819985.1 (*Aeromonas media* CECT 4232^T^), NZ_CP027856.1/GCF_003015165.1 (*Aeromonas rivipollensis* KN-Mc-11 N1), NZ_UAPT00000000.1/GCF_900445115.1 (*Aeromonas salmonicida* NCTC 12959^T^), NZ_CDDK00000000.1/GCF_000820225.1 (*Aeromonas veronii* CECT 4257^T^), NC_012691.1/GCF_000023065.1 (*Tolumonas auensis* DSM 9187^T^), NZ_AZUK00000000.1/GCF_000527035.1 (*Tolumonas lignilytica* BRL6-1^T^), and NZ_JACHGR000000000.1/GCF_014202415.1 (*Tolumonas osonensis* DSM 22975^T^).

## 3 Results

### 3.1 Bacterial identification and phylogeny

Whole-cell MALDI-TOF MS successfully confirmed all 52 presumptive *Aeromonas* spp. isolates under study to the genus level with high confidence (Bruker MALDI-TOF score ≥ 2.0 and consistency category A or B).

Whole genome sequencing followed by Similar Genome Finder- and ANI calculator-based analyses reliably identified 49 of the 52 *Aeromonas* isolates at the species level (OrthoANIu value between query genome and NCBI reference genome >95%). Species results are summarized in Table 2. Detailed information is given in Supplementary Table S3. *Aeromonas* isolates from German patients were classified to seven different species. The three most prevalent *Aeromonas* species were *A. veronii* (*n* = 18; 35%), *A. caviae* (*n* = 17; 33%), and *A. hydrophila* (*n* = 9; 17%) (Table 3). Less frequently, *A. salmonicida* (*n* = 2; 4%), *A. dhakensis*, *A. bestiarum*, and *A. encheleia* (*n* = 1 each; 2% each) were detected. The latter two isolates, 21-AL00126 and 21-AL00125, were isolated from one patient. For three isolates (21-AL00040, 21-AL00070, and 21-AL00079), only ANI values below the cut-off score were obtained and they were therefore designated as “*Aeromonas* sp.” These isolates showed the highest similarity to the species *A. media*/*A. rivipollensis* with OrthoANIu values between 93.81 and 94.55%.

**Table 2:** *Aeromonas* spp. strains (*n* = 52) from German patients (*n* = 51) analyzed in this study.

Strain	Species*^a^*	Year of isolation	Source of isolation	Patient’s age group (years)	Patient’s gender*^b^*	Geographical origin*^c^*	Type of infection	Source code*^e^*
Intestinal (*n* = 30)								
21-AL00012	*A. caviae*	2016	Stool	>60	M	MV*	Diarrhea	C-G-cr-int
21-AL00013	*A. veronii*	2016	Stool	>60	F	MV***	Diarrhea	C-G-int
21-AL00015	*A. caviae*	2016	Stool	>60	F	MV*	Diarrhea	C-G-cr-int
21-AL00021	*A. caviae*	2017	Stool	25–60	F	MV*	Gastroenteritis	C-G-cr-int
21-AL00032	*A. salmonicida*	2017	Stool	25–60	M	TH**	Diarrhea	C-G-ncr-int
21-AL00040	*Aeromonas* sp.	2017	Stool	25–60	F	TH**	Enteritis	C-G-ncr-int
21-AL00052	*A. veronii*	2018	Stool	>60	F	BB**	Diarrhea	C-G-ncr-int
21-AL00066	*A. caviae*	2018	Stool	11–24	M	TH**	Enteritis	C-G-ncr-int
21-AL00070	*Aeromonas* sp.	2018	Stool	>60	M	MV*	Diarrhea	C-G-cr-int
21-AL00072	*A. caviae*	2018	Stool	>60	F	BB**	Diarrhea	C-G-ncr-int
21-AL00078	*A. caviae*	2018	Stool	>60	F	BB**	Diarrhea	C-G-ncr-int
21-AL00079	*Aeromonas* sp.	2018	Stool	25–60	M	BB**	Gastroenteritis	C-G-ncr-int
21-AL00081	*A. veronii*	2018	Stool	≤10	M	BB**	Gastroenteritis	C-G-ncr-int
21-AL00083	*A. veronii*	2018	Stool	>60	M	BB**	Enteritis	C-G-ncr-int
21-AL00084	*A. veronii*	2018	Stool	25–60	F	BB**	Diarrhea	C-G-ncr-int
21-AL00085	*A. veronii*	2018	Stool	11–24	F	BB**	Appendicitis	C-G-ncr-int
21-AL00105	*A. caviae*	2019	Stool	>60	M	BB**	Diarrhea	C-G-ncr-int
21-AL00110	*A. caviae*	2019	Stool	>60	F	BB**	Diarrhea	C-G-ncr-int
21-AL00111	*A. caviae*	2019	Stool	25–60	M	MV*	Gastroenteritis	C-G-cr-int
21-AL00113	*A. caviae*	2019	Stool	≤10	F	MV*	Gastroenteritis	C-G-cr-int
21-AL00115	*A. hydrophila*	2019	Stool	25–60	F	BB**	Gastroenteritis	C-G-ncr-int
21-AL00118	*A. caviae*	2019	Stool	≤10	M	MV*	Gastroenteritis	C-G-cr-int
21-AL00128	*A. veronii*	2019	Stool	>60	F	BB**	Diarrhea	C-G-ncr-int
21-AL00131	*A. veronii*	2019	Stool	>60	F	BB**	Diarrhea	C-G-ncr-int
21-AL00133	*A. veronii*	2019	Stool	25–60	M	MV*	Bloody stool	C-G-cr-int
21-AL00134	*A. veronii*	2019	Stool	25–60	M	MV*	Enteritis	C-G-cr-int
21-AL00136	*A. veronii*	2019	Stool	>60	M	NW**	Diarrhea	C-G-ncr-int
21-AL00139	*A. caviae*	2019	Stool	25–60	F	MV*	Gastroenteritis	C-G-cr-int
21-AL00142	*A. veronii*	2019	Stool	>60	F	BB**	Diarrhea	C-G-ncr-int
21-AL00143	*A. veronii*	2019	Stool	25–60	M	BB**	Enteritis	C-G-ncr-int
Extraintestinal (*n* = 22)								
21-AL00018	*A. caviae*	2017	Abdominal swab (intraoperative)	>60	F	MV***	Gangrenous cholecystitis, diarrhea	C-G-ext
21-AL00065	*A. caviae*	2018	Urine	>60	F	TH**	Pyelonephritis	C-G-ncr-ext
21-AL00068	*A. veronii*	1996	Blood culture	>60	M	NW**	Bile duct obstruction with fever, chills, and jaundice	C-G-ncr-ext
21-AL00075	*A. veronii*	2018	Blood culture	>60	M	BB**	Diarrhea, septicemia	C-G-ncr-ext
21-AL00076	*A. hydrophila*	2018	Wound swab	>60	M	BB**	Wound infection	C-G-ncr-ext
21-AL00080	*A. veronii*	2018	Blood culture	>60	M	BB**	Wound infection, septicemia	C-G-ncr-ext
21-AL00082	*A. hydrophila*	2018	Bronchial secretion	25–60	M	BB**	Pneumonia	C-G-ncr-ext
21-AL00086	*A. caviae*	2018	Urine	25–60	F	BB**	Urinary tract infection	C-G-ncr-ext
21-AL00088	*A. hydrophila*	2018	Wound swab	>60	M	BB**	Wound infection	C-G-ncr-ext
21-AL00091	*A. hydrophila*	2019	Wound swab	25–60	M	MV***	Wound infection	C-G-ext
21-AL00095	*A. caviae*	2019	Wound swab	>60	F	HE**	Wound infection	C-G-ncr-ext
21-AL00096	*A. salmonicida*	2019	Wound swab	25–60	F	NW**	Wound infection	C-G-ncr-ext
21-AL00103	*A. caviae*	2019	Blood culture	>60	M	NW**	Septicemia	C-G-ncr-ext
21-AL00116	*A. hydrophila*	2019	Wound swab	>60	M	NW**	Wound infection	C-G-ncr-ext
21-AL00124	*A. veronii*	2019	Puncture specimen of the lower leg	25–60	M	NW**	Wound infection	C-G-ncr-ext
21-AL00125	*A. encheleia*	2019	Knee swab	25–60	M	NW**	Bursitis*^d^*	C-G-ncr-ext
21-AL00126	*A. bestiarum*	2019	Knee swab	25–60	M	NW**	Bursitis*^d^*	C-G-ncr-ext
21-AL00130	*A. hydrophila*	2019	Wound swab	>60	F	BB**	Abscess in the area of adnexa	C-G-ncr-ext
21-AL00132	*A. dhakensis*	2019	Wound swab	≤10	F	NW**	Abscess at the bottom of the foot	C-G-ncr-ext
21-AL00137	*A. hydrophila*	2019	Blood culture	25–60	F	MV*	Cholangitis, including bacteremia	C-G-cr-ext
21-AL00140	*A. hydrophila*	2019	Wound swab	25–60	M	MV*	Wound infection	C-G-cr-ext
21-AL00141	*A. veronii*	2019	Wound swab	25–60	M	BB**	Wound infection	C-G-ncr-ext

a Whole genome sequence (WGS)-based species results were obtained as part of this study and are explained in detail in Supplementary Table S3.

b F, Female; M, Male.

c German federal states are given. BB, Brandenburg; HE, Hesse; MV, Mecklenburg-Western Pomerania; NW, North Rhine-Westphalia; TH, Thuringia. The coastal typology of the finding region (coastal or non-coastal region) is indicated by asterisks. In accordance with the European Union’s recommendations, a region was defined as a coastal region if it met one of the following three criteria: it is (i) a region that borders the sea, (ii) a region in which more than 50% of its population live within 50 km from the coastline, and (iii) the region of Hamburg [Eurostat (Statistical Office of the European Union), 2019]. *Coastal region; **Non-coastal region; ***The patient’s place of residence (region) is unknown. Information on coastal typology is not available.

d Isolates from the same patient.

e C, clinical; G, Germany; cr, coastal region; ncr, non-coastal region; int, intestinal; ext, extraintestinal. The superscript letter that introduces the explanations in italics.

**Table 3:** Distribution of *Aeromonas* spp. recovered from patients (*n* = 51) with domestically acquired *Aeromonas* spp. infections by type of infection, Germany, 2016–2019*^a^*.

Type of infection	No. of *Aeromonas* spp. isolates*^g^*								
	Total	*A. veronii*	*A. caviae*	*A. hydrophila*	*A. salmonicida*	*A. dhakensis*	*A. bestiarum*	*A. encheleia*	*Aeromonas* sp.
All	52 (100%)	18 (35%)	17 (33%)	9 (17%)	2 (4%)	1 (2%)	1 (2%)	1 (2%)	3 (6%)
Intestinal	30	13	12	1	1	0	0	0	3
(Gastro-)Enteritis*^b^*	28	11	12	1	1	0	0	0	3
Appendicitis	1	1	0	0	0	0	0	0	0
Others*^c^*	1	1	0	0	0	0	0	0	0
Extraintestinal	22	5	5	8	1	1	1	1	0
Wound infection	9	2	1	5	1	0	0	0	0
Septicemia*^d^*	3	2	1	0	0	0	0	0	0
Cholangitis/Cholecystitis*^e^*	2	0	1	1	0	0	0	0	0
Pneumonia	1	0	0	1	0	0	0	0	0
Pyelonephritis	1	0	1	0	0	0	0	0	0
Urinary tract infection	1	0	1	0	0	0	0	0	0
Bursitis	2*^h^*	0	0	0	0	0	1	1	0
Others*^f^*	3	1	0	1	0	1	0	0	0

a One infection was acquired in 1996.

b (Gastro-)Enteritis includes all cases of gastroenteritis, enteritis, and reported diarrhea.

c Bloody stool. Type of infection not specified.

d Septicemia includes: (i) septicemia with previous diarrhea (*n* = 1): *A. veronii* (*n* = 1), (ii) septicemia associated with wound infection (*n* = 1): *A. veronii* (*n* = 1), and (iii) septicemia (*n* = 1): *A. caviae* (*n* = 1).

e Cholangitis/Cholecystitis includes: (i) cholangitis including bacteremia (*n* = 1): *A. hydrophila* (*n* = 1) and (ii) gangrenous cholecystitis with additional diarrhea (*n* = 1): *A. caviae* (*n* = 1).

f Others include: (i) bile duct obstruction with fever, chills, and jaundice followed by *Aeromonas* detection in blood cultures (*n* = 1): *A. veronii* (*n* = 1), (ii) abscess in the area of adnexa (*n* = 1): *A. hydrophila* (*n* = 1), and (iii) abscess at the bottom of the foot (*n* = 1): *A. dhakensis* (*n* = 1).

g Detailed information on the whole genome sequence (WGS)-based typed *Aeromonas* spp. isolates is given in Table 2.

h Isolates from the same patient. The superscript letter that introduces the explanation in italics.

API 20 E testing confirmed that all identified *A. veronii* isolates could be assigned to the biovar *sobria* (Martin-Carnahan and Joseph, 2015).

A phylogenetic study between the 52 clinical *Aeromonas* isolates and seven NCBI reference strains was performed using Panaroo and IQ-TREE. The genomes of three reference strains of the genus *Tolumonas* that belongs to the family *Aeromonadaceae* were included as outgroup.

**Figure 1:**
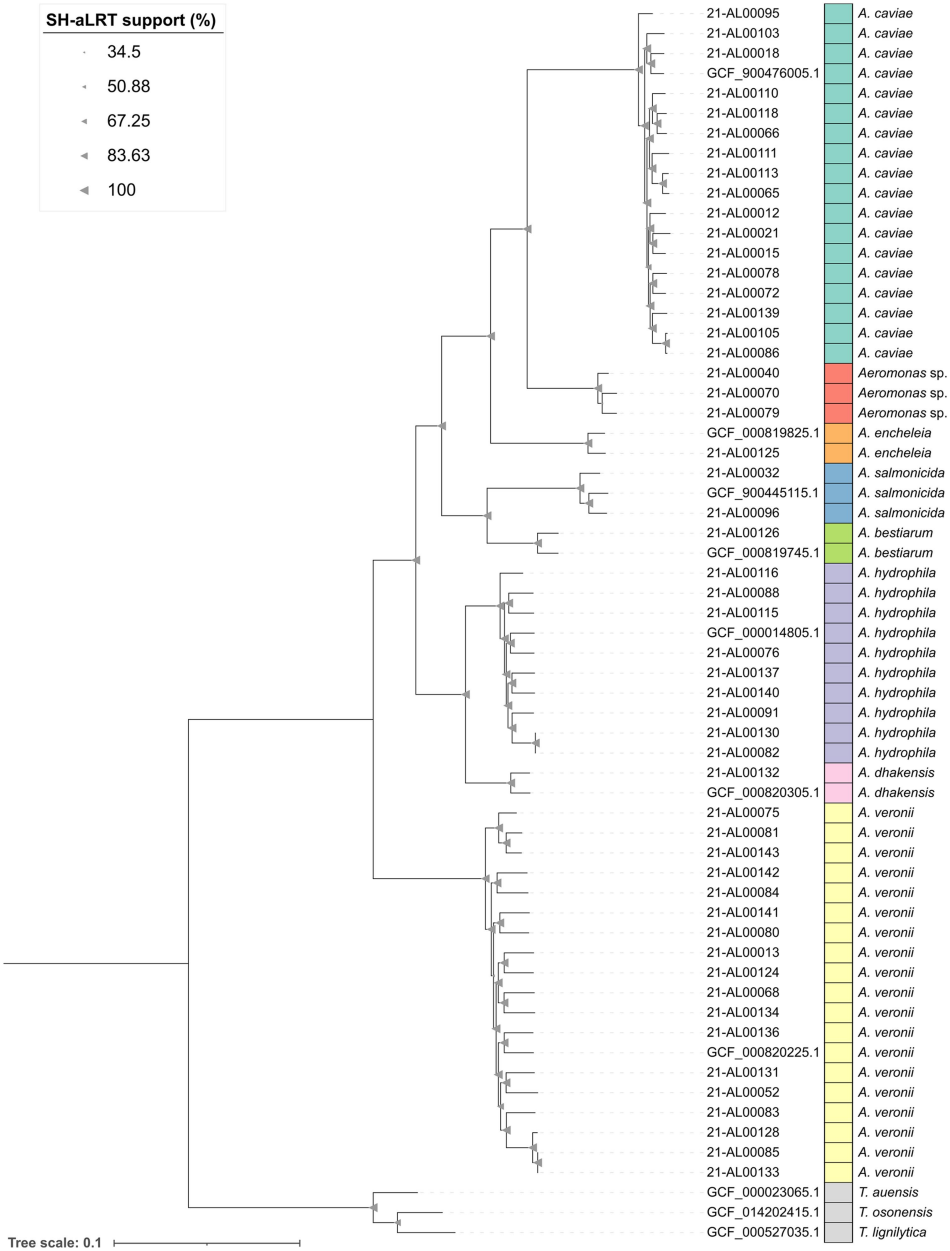
Maximum-likelihood phylogenetic tree of the 52 clinical *Aeromonas* isolates from this study as well as seven representative *Aeromonas* reference strains and three reference strains of the genus *Tolumonas* from NCBI generated by using Panaroo v1.3.4 and IQ-TREE v2.2.0. The tree was visualized in iTOL v6 and rooted on the *Tolumonas* outgroup. Branch support values (SH-aLRT) were calculated in 1,000 replicates and are indicated as triangles on the branches. The size of the triangle correlates with the support value. The scale bar represents the average number of substitutions per site.

The analysis of the assemblies by Panaroo revealed that all 59 *Aeromonas* and three *Tolumonas* strains shared a core genome of 2,258 genes (after filtering). The alignment length of the filtered core genome was approximately 2.3 Mbp. The maximum-likelihood phylogenetic tree generated based on this alignment by IQ-TREE was visualized in iTOL and is shown in Figure 1.

The results of the phylogenetic study were in accordance with the ANI (average nucleotide identity) results. The maximum-likelihood tree showed the grouping of the 52 clinical *Aeromonas* spp. genomes into eight clusters (Figure 1, bars dark green to yellow). *Aeromonas* isolates exhibiting clear ANI-supported species results formed species-specific clusters with the corresponding type strains. One cluster (red in Figure 1) was formed by the three “*Aeromonas* sp.” isolates.

The human clinical *A. dhakensis* isolate (strain 21-AL00132) was related to the *A. hydrophila* cluster. The clinical *A. encheleia* isolate (strain 21-AL00125) was closer related to the *A. caviae* cluster. The *A. encheleia*/*A. caviae* cluster grouped together with the clinical *A. bestiarum* isolate (strain 21-AL00126) and the two clinical *A. salmonicida* isolates (strains 21-AL00032 and 21-AL00096) (Figure 1).

Interestingly, clinical *A. veronii* isolates and *A. hydrophila* isolates showed a higher genomic divergence than clinical *A. caviae* isolates based on the length of the branches within the respective clusters.

**Figure 2:**
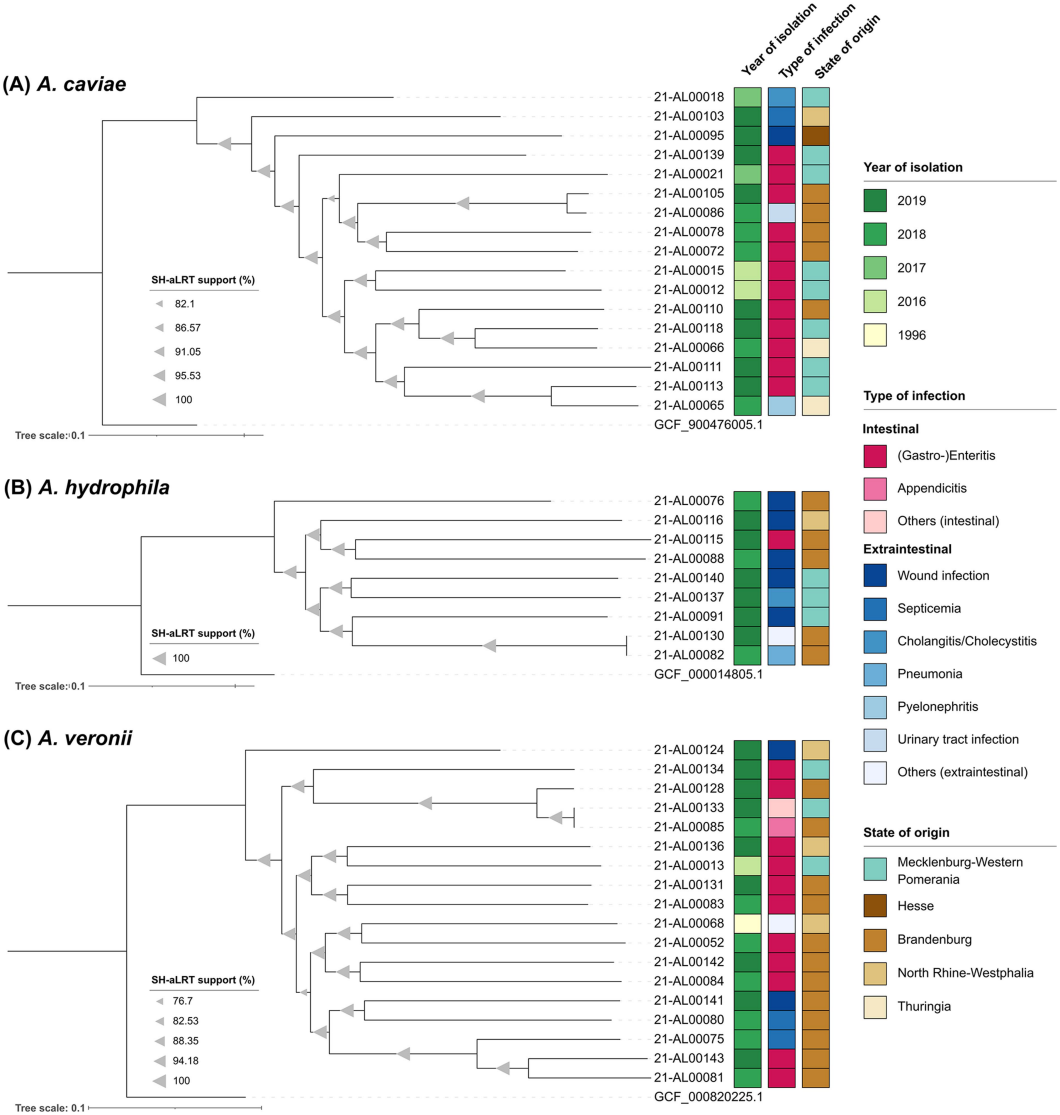
Maximum-likelihood phylogenetic trees of the three main *Aeromonas* species from this study generated using the snippySnake pipeline v1.3.0 in combination with IQ-TREE v2.2.0, visualized in iTOL v6 and manually rooted to the respective NCBI reference genome. **(A)** *Aeromonas caviae* phylogenetic tree based on 217,564 SNP positions. **(B)** *Aeromonas hydrophila* phylogenetic tree based on 316,836 SNP positions. **(C)** *Aeromonas veronii* phylogenetic tree based on 395,268 SNP positions. Branch support values (SH-aLRT) were calculated in 1,000 replicates and are indicated as triangles on the branches. The size of the triangle correlates with the support value. The scale bar represents the average number of substitutions per SNP position.

For a more detailed look at the phylogenetic relationship of isolates belonging to the three main *Aeromonas* species from this study (*A. caviae*, *A. hydrophila*, and *A. veronii*), maximum-likelihood-based phylogenetic trees were inferred from the respective core SNPs. The maximum-likelihood phylogenetic trees generated based on this alignment by IQ-TREE were visualized in iTOL and are shown in Figure 2. The corresponding SNP distance matrices are provided in Supplementary Table S4.

All sequenced *A. caviae* genomes were analyzed in a SNP analysis with the genome of *A. caviae* strain NCTC 12244 (GCF_900476005.1) as reference. In total, 3,402,863 positions (corresponding to approximately 74% of the reference genome) were used in the analysis and the number of SNPs between the isolates varied between 5,672 and 60,127. The majority of German clinical *A. caviae* isolates distinctly differed from each other [SNP differences more than 30,000 (average 52,759)]. Only two Brandenburgian isolates (strains 21-AL00105 and 21-AL00086) were closer related (SNP difference around 5,700). The same was true for a Thuringian isolate (strain 21-AL00065) and an isolate from Mecklenburg-Western Pomerania (strain 21-AL00113) (approximately 22,500 SNPs). The SNP-derived tree reproduces these genomic differences (Figure 2A).

Similar observations were made for the remaining two species (Figures 2B,C). To characterize the relationship between the clinical *A. hydrophila* isolates further, a SNP analysis with the genome of *A. hydrophila* strain ATCC 7966 (GCF_000014805.1) as reference was performed. In total, the percentage of the reference genome covered by all isolates was 84% (3,997,565 positions in all analyzed genomes). The number of SNPs between the isolates varied between 26 and 107,960. With the exception of two isolates from Brandenburg (strains 21-AL00130 and 21-AL00082; SNP difference below 30), all clinical *A. hydrophila* isolates clearly differed from each other [SNP differences more than 97,500 (average 100,860)] (Supplementary Table S4). The 18 *A. veronii* genomes were analyzed for their phylogenetic relationship using SNP analysis with the genome of *A. veronii* strain CECT 4257 (GCF_000820225.1) as reference. In total, 3,343,295 positions (corresponding to approximately 74% of the reference genome) were used of the investigated genomes and the number of SNPs between the isolates varied between 78 and 105,205. A close relatedness was exclusively observed for the isolates 21-AL00133 and 21-AL00085 (SNP difference below 80) that were related to the Brandenburgian isolate 21-AL00128 (SNP difference around 18,000). The differences between the genomes of the remaining clinical *A. veronii* isolates were more than 50,000. The average SNP difference between two clinical isolates was 99,603 (Supplementary Table S4).

All three SNP-derived maximum-likelihood phylogenetic trees split into several clusters (Figures 2A–C). However, no clear pattern was observed in reference to the year of isolation, the associated type of infection, or the geographical origin of the isolates. Even at a larger distance from the species node, *A. veronii* isolates of different geographical origins occasionally grouped together (e.g., strain 21-AL00133 isolated from a patient in a coastal region of Mecklenburg-Western Pomerania and strain 21-AL00085 from a non-coastal region of the German state Brandenburg). Similar phylogenetic subclusters were noted in the *A. hydrophila* and the *A. caviae* group.

### 3.2 Patients’ characteristics and types of infection

Since no travel history was documented for any of the 51 patients, it can be assumed that all *Aeromonas* spp. infections were acquired in Germany.

Table 4 shows the type of infection related to age group, gender, and geographical origin of the patients. Domestically acquired *Aeromonas* spp. infections occurred across all age groups (age range: 9 months to 90 years). Children and young adults were rarely affected. Most infections were observed in elderly people over 60 years old. Gender ratio (male: female) was 1.13 (27/24) among all patients. Extraintestinal infections were observed more frequently in male patients [13 out of 27 (male) vs. 8 out of 24 (female)]. However, the statistical analysis demonstrated that the described difference was not significant (*p* > 0.05, Fisher’s exact test). Overall, cases of disease occurred in both coastal and non-coastal regions, with three times as many cases recorded in inland areas. Detailed information on the route of transmission was not available.

**Table 4:** Characteristics of patients with domestically acquired *Aeromonas* spp. infections by type of infection, Germany, 2016–2019*^a^* (*n* = 51).

Patients’ characteristics	Type of infection*^d^*		
	Total (*n* = 51)	Intestinal (*n* = 30)	Extraintestinal (*n* = 21)
Age group (years)			
≤ 10	4	3	1
11–24	2	2	0
25–60	20	11	9
> 60	25	14	11
Gender*^b^*			
F	24	16	8
M	27	14	13
Geographical origin*^c^*			
Coastal region	12	10	2
Non-coastal region	36	19	17
Unknown	3	1	2

a One infection was acquired in 1996.

b F, Female; M, Male.

c In accordance with the European Union’s recommendations, a region was defined as a coastal region if it met one of the following three criteria: it is (i) a region that borders the sea, (ii) a region in which more than 50% of its population live within 50 km from the coastline, and (iii) the region of Hamburg [Eurostat (Statistical Office of the European Union), 2019]. Detailed information on the geographical region is given in Table 2.

d Detailed information on the type of infection is given in Tables 2, 3. The superscript letter that introduces the explanation in italics.

The distribution of the types of infection is shown in Table 3. A total of 30 patients (59%) contracted intestinal infections. Among the patients suffering from extraintestinal infections (*n* = 21; 41%), nine (43%) presented with wound infections, three (14%) with septicemia, and two (10%) with cholangitis/cholecystitis. Further, single cases of pneumonia, pyelonephritis, and urinary tract infection were observed. One patient presenting with bursitis was suffering from a co-infection with two *Aeromonas* species, *A. encheleia* and *A. bestiarum*.

For 13 of the 51 patients (25%; average age: 60 years), severe courses of disease were reported. Several patients required hospitalization (nine reported cases, 18%). Septicemia exclusively occurred in elderly patients (average age: 77 years), two of whom had an underlying oncological or cardiovascular disease. For the third septicemia patient, as for the majority of the remaining 48 patients, no information on underlying diseases was available.

As presented in Table 3, 30 intestinal infections were primarily diagnosed with *A. veronii* (*n* = 13; 43%) or *A. caviae* (*n* = 12; 40%). Extraintestinal infections (22 isolates) were mainly associated with *A. hydrophila* (*n* = 8; 38%) followed by *A. veronii* and *A. caviae* (*n* = 5 each; 24% each).

### 3.3 Presence/absence of virulence genes

Whole genome sequencing revealed that of the 288 *Aeromonas* virulence genes examined in this study, in total 261 genes were detected with varying frequencies (see Table 1 and Supplementary Table S2).

#### 3.3.1 Motility genes

One hundred and nineteen flagella-associated genes involved in motility were considered and 101 genes were identified in the clinical isolates. Detailed information on the presence/absence of these virulence genes in the *Aeromonas* spp. isolates is given in Supplementary Table S2.

#### 3.3.2 Adherence genes encoding pili

The presence of a number of genes coding for adherence factors was investigated. 57 genes of in total 58 adherence genes were detected in the clinical isolates. These genes comprised all analyzed Flp type IV pili-coding genes (*flp1*, *flpA*-*flpL*), MSHA type IV pili-coding genes (*mshA*-*mshG*, *mshI* and *mshI1*, *mshJ*-*mshQ*), and type I pili-coding genes (*fimA*, *fimC*-*fimF*). Moreover, almost all analyzed Tap type IV pilus system-associated genes (*ASA_RS18105*, *tapB*-*tapD*, *tapF*, *tapM*-*tapQ*, *tapT*-*tapW*, *tapY1*, and *tapY2*, *tppA*-*tppF*) were found. Only the *tapA* gene encoding a pilin subunit (Pepe et al., 1996) was not detected in the clinical isolates.

#### 3.3.3 Effector delivery system-associated genes encoding secretion systems T2SS, T3SS, and T6SS

Of the 86 genes encoding effector delivery systems and associated effectors investigated in this study, 78 genes were present in the clinical isolates. This included all analyzed Exe T2SS-coding genes (*exeA*-*exeN*) and T6SS-associated genes (*AHA_RS09305*/*ASA_RS12290*, *atsA*-*atsD*, *atsG*-*atsL*, *atsP*, *atsQ*, *atsS*, *clpB*, *dotU*, *hcp*, *hcp1*, *vasH*, *vasK*, *vgrG1*-*vgrG3*, *vipA*, and *vipB*) as well as the majority of the studied T3SS-associated genes (*acr1*, *acr2*, *acrG*, *acrH*, *acrR*, *acrV*, *aopB*, *aopD*, *aopN*, *aopX*, *ascB*-*ascL*, *ascN*-*ascV*, *ascX*, *ascY*, *exsA*-*exsE*, *sycX*, and *ABH06556*).

#### 3.3.4 Exotoxin genes

Exotoxin encoding genes play a role in pathogenicity. We studied the presence of 15 exotoxin genes and the analysis revealed that all genes were found in the clinical isolates. The exotoxin genes enclosed the aerolysin A-coding gene (*aerA*) and genes encoding an aerolysin-like hemolysin (*AAC44637*, *AAN77507*, and *ABJ52834*), RtxA-associated genes (*rtxA*-*rtxE*, *rtxH*), the hemolysin III gene and the thermostable hemolysin gene as well as *ahh1*, *ast* and *hlyA*.

#### 3.3.5 Genes associated with nutritional/metabolic factors

The presence of 10 genes associated with nutritional or metabolic factors playing a role in virulence was investigated. All genes were found in the human clinical isolates. In addition to the *amoA* gene coding for a biosynthesis protein of amonabactin (phenolate siderophore) (Barghouthi et al., 1989; Barghouthi et al., 1991), this encompassed all analyzed genes encoding the heme uptake system (*ASA_RS16540*, *ASA_RS16545*, *ASA_RS16550*, *ASA_RS16555*, *ASA_RS16570*, *ASA_RS16575*, *ASA_RS16580*, *hutX*, and *hutZ*).

### 3.4 Assessment of the occurrence of virulence factors with regard to infection type and species

Sixteen main virulence factors involved in the categories adherence, effector delivery systems, exotoxins, and nutritional/metabolic factors of the VFDB were considered in this study. Based on the collected WGS data, their presence was assessed and varying frequencies regarding infection type (intestinal/extraintestinal) and *Aeromonas* species (*A. caviae*, *A. hydrophila*, and *A. veronii*) were observed. The results are summarized in Supplementary Table S5. Detailed information is given in Supplementary Table S2. In Supplementary Table S5, the geographical origin of the clinical isolates is also shown regarding differences in the occurrence for coastal and non-coastal regions.

#### 3.4.1 Adherence

Tap type IV pili and MSHA type IV pili were probably completely or partially present in all and nearly all (98%) of the 52 *Aeromonas* spp. isolates, respectively. Genes for type I pili (T1P) and Flp type IV pili were only detected in 33% or 17% of the isolates, respectively.

When the isolates were divided according to the type of infection, only for type I pili genes a considerable difference was observed. T1P genes were more present in extraintestinal isolates (50%, 11 out of 22 isolates) than in intestinal isolates (20%, six out of 30 isolates). The statistical analysis showed that the more frequent occurrence of T1P genes in extraintestinal isolates was significant (*p* = 0.0360, Fisher’s exact test).

Subdivision of the isolates by species revealed clear differences in the frequency of occurrence of both T1P and Flp type IV pili, with type I pili being present in all *A. hydrophila* isolates (9 out of 9), 17% of the *A. veronii* isolates (three out of 18), and only 6% of the *A. caviae* isolates (one out of 17). The observed differences to *A. hydrophila* isolates were statistically highly significant (*p* < 0.001, Fisher’s exact test). The presence of type I pili genes has been reported in most *A. hydrophila* strains, but their role in pathogenicity has been questioned (Gonçalves Pessoa et al., 2019). Flp type IV pili genes were identified in 33% of the *A. veronii* isolates (six out of 18), while they were absent in all *A. hydrophila* (*n* = 9) and *A. caviae* (*n* = 17) isolates examined. However, only the described difference between the *A. veronii* isolates and the *A. caviae* isolates was statistically significant (*p* = 0.0191, Fisher’s exact test).

#### 3.4.2 Effector delivery systems

The *Aeromonas* type II secretion system (Exe T2SS) was probably completely present in all of the 52 *Aeromonas* spp. isolates investigated in this study. In addition, genes for the type III secretion system (T3SS) and the type VI secretion system (T6SS) were detected in 25 and 52% of the isolates, respectively.

When the isolates were differentiated by type of infection, 41% (9 out of 22) of the extraintestinal isolates probably possessed a T3SS and 77% (17 out of 22) a T6SS. In contrast, only 13% (4 out of 30) of the intestinal isolates studied had T3SS genes and 33% (10 out of 30) had T6SS genes. The statistical analysis demonstrated that the more frequent occurrence of the T3SS or the T6SS in extraintestinal isolates was significant to very significant (*p* = 0.0492 (T3SS), *p* = 0.0023 (T6SS), Fisher’s exact test).

Grouping of the isolates by species also revealed distinct differences in the frequency of occurrence of T3SS and T6SS. Type VI secretion system genes were detected in *A. hydrophila* isolates, *A. veronii* isolates, and *A. caviae* isolates. However, the type III secretion system was missing in all *A. caviae* isolates examined (*n* = 17). With a frequency of occurrence of 100% (9 out of 9) or 67% (6 out of 9), the T6SS and T3SS were most frequently detected in *A. hydrophila* isolates. Of the *A. veronii* isolates studied, 33% (6 out of 18) were T6SS-positive and with the same frequency T3SS-positive. Likewise, T6SS genes were found in 35% (6 out of 17) of the *A. caviae* isolates. The observed differences to *A. hydrophila* isolates were statistically significant (except the T3SS difference *A. veronii*-*A. hydrophila*, *p* > 0.05, Fisher’s exact test).

#### 3.4.3 Exotoxins

Furthermore, assessment of virulence factors based on WGS data indicated that hemolysin HlyA and hemolysin III occurred in all of the 52 *Aeromonas* spp. isolates. Beyond that, the thermostable hemolysin was detected in nearly all isolates (98%). In addition, 58% of the isolates were considered aerolysin-positive due to the presence of the aerolysin A gene (*aerA*; 35%) and/or an aerolysin-like hemolysin gene (ALH gene; 23%). The genes for the extracellular hemolysin AHH1, the heat-stable cytotonic enterotoxin Ast, and RtxA were found in 25, 21, and 12% of the isolates, respectively.

When the isolates were categorized according to the type of infection, 50% (11 out of 22) of the extraintestinal isolates probably possessed the extracellular hemolysin AHH1, 45% (10 out of 22) the heat-stable cytotonic enterotoxin Ast, and 23% (5 out of 22) RtxA. In contrast, only 7% (2 out of 30) of the intestinal isolates examined were AHH1-positive and 3% each (1 out of 30) were Ast-positive and RtxA-positive, respectively. The statistical analysis showed that the more frequent occurrence of AHH1 or Ast in extraintestinal isolates was highly significant (*p* < 0.001, Fisher’s exact test).

Subdividing the isolates by species revealed not only considerable differences in the frequency of occurrence of genes for AHH1, Ast, and RtxA but also for aerolysin, with aerolysin A and/or aerolysin-like hemolysin being present in both *A. veronii* and *A. hydrophila* isolates. In contrast, these hemolysin genes were absent in all *A. caviae* isolates (*n* = 17). With a frequency of occurrence of 100% (18 out of 18) and 67% (6 out of 9), aerolysin was detected more frequently in *A. veronii* isolates than in *A. hydrophila* isolates. The observed differences to *A. caviae* isolates were statistically highly significant (*p* < 0.001, Fisher’s exact test). Genes encoding exotoxins AHH1, Ast, and RtxA were found exclusively in *A. hydrophila* isolates, with frequencies of occurrence of 100% (*ahh1*, *ast*) and 33% (RtxA-associated genes).

#### 3.4.4 Nutritional/metabolic factors

The heme uptake system was probably completely or partially present in all of the 52 *Aeromonas* spp. isolates investigated in this study. Additionally, amonabactin was detected in over half of the isolates (63%).

When the isolates were differentiated by type of infection, 77% (17 out of 22) of the extraintestinal isolates possessed amonabactin, whereas only about half of the intestinal isolates studied (53%; 16 out of 30) had this virulence factor. However, the statistical analysis demonstrated that the more frequent occurrence of amonabactin in extraintestinal isolates was not significant (*p* > 0.05, Fisher’s exact test).

Subdivision of the isolates by species also revealed clear differences in the occurrence of amonabactin gene, with *amoA* being detected in all *A. caviae* (*n* = 17) and *A. hydrophila* (*n* = 9) isolates, while it was missing in the *A. veronii* isolates (*n* = 18). The described differences to *A. veronii* isolates were statistically highly significant (*p* < 0.001, Fisher’s exact test).

### 3.5 Determination of virulence factor profiles and cluster analysis

**Figure 3:**
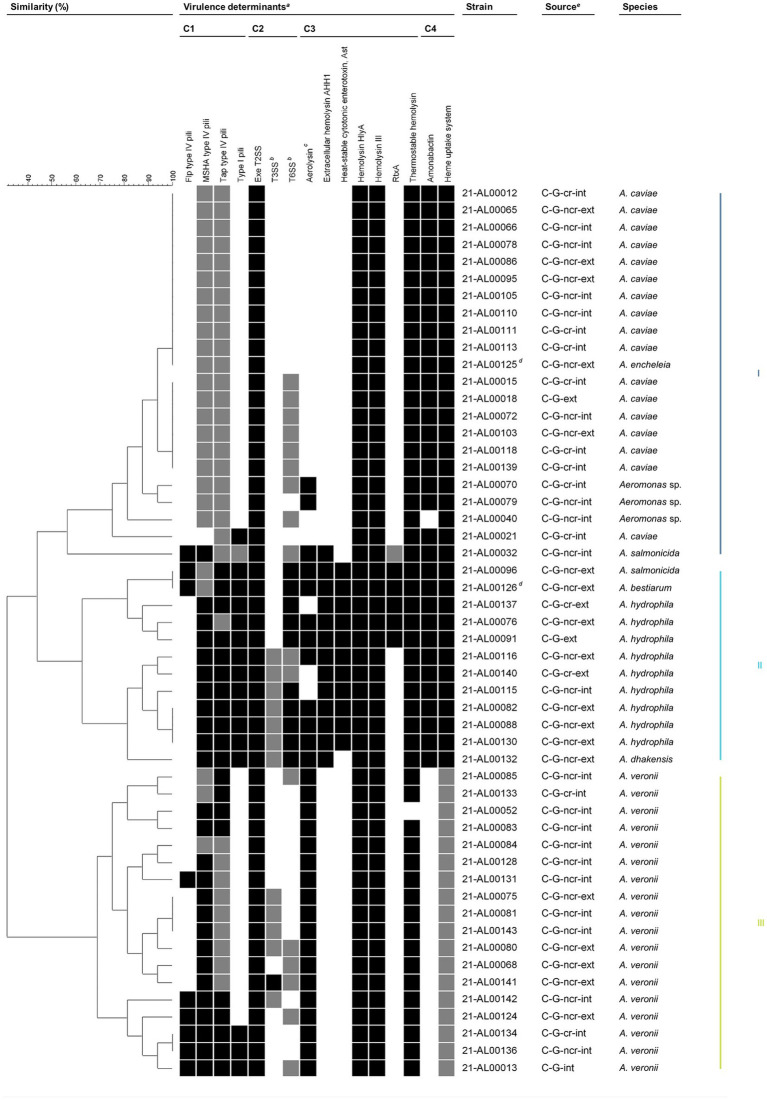
Grouping of *Aeromonas* spp. isolates from German patients based on virulence traits. Similarity patterns were determined by Complete linkage using categorical (values) similarity coefficient of ternary data (BioNumerics v7.6.3, Applied Maths NV, Sint-Martens-Latem, Belgium). Colored bars on the right indicate the main clusters present based on a similarity level of 55%. *^a^*Virulence factors are sorted by functional category: adherence (C1), effector delivery systems (C2), exotoxins (C3), and nutritional/metabolic factors (C4). Flp, Fimbrial low-molecular weight protein; MSHA, Mannose-sensitive hemagglutinin; RtxA, Repeat in toxin A; Tap, Type IV *Aeromonas* pilus; T2SS, Type II secretion system; T3SS, Type III secretion system; T6SS, Type VI secretion system. The color intensity of the boxes indicates the presence of the virulence factor: probably completely present (black), partially present (gray), and absent (white). In general, a virulence factor was considered to be probably completely present if ≥80% of the genes examined in this study were detected. Further details are given in Table 1 and Supplementary Table S2 (sheets “Summary Table” and “Assessment Key”). *^b^*Including secreted effectors. *^c^*Strains were considered aerolysin-positive if aerolysin A (AerA) and/or an aerolysin-like hemolysin (ALH) were present. *^d^*Isolates from the same patient. *^e^*C, clinical; G, Germany; cr, coastal region; ncr, non-coastal region; int, intestinal; ext, extraintestinal.

Presence/absence data were used to create virulence factor profiles and determine similarity patterns between the human clinical *Aeromonas* spp. isolates by the complete linkage method (Figure 3).

A total of 31 different virulence factor profiles were obtained. The comparative profile analysis revealed that a basic set of virulence factors is present in nearly all clinical isolates. The observed seven virulence factors were: adherence factors [MSHA type IV pili (complete (c)/partial (p)), Tap type IV pili (c/p)], effector delivery systems [Exe T2SS (c)], exotoxins [hemolysin HlyA (c), hemolysin III (c), thermostable hemolysin (c)], and nutritional/metabolic factors [heme uptake system (c/p)]. Beyond that, varying additional virulence traits from one or more of the four functional trait categories are present in all of these clinical isolates. Based on a similarity level of 55%, three main clusters emerged.

#### 3.5.1 Cluster III: *Aeromonas veronii*

Cluster III contained all human clinical *A. veronii* isolates examined in this study.

The *A. veronii* isolates exhibited virulence patterns that included seven to 11 virulence traits. The majority of the clinical *A. veronii* isolates (94%) were characterized by the seven basic virulence factors mentioned above and aerolysin (c). In 33% of the clinical *A. veronii* isolates, one additional virulence factor (Flp type IV pili, T3SS, or T6SS) was probably completely or partially present, while in 39% of the isolates, combinations of two or more additional virulence traits (Flp type IV pili, type I pili, T3SS, T6SS) were observed. The T3SS and the T6SS were detected in 60% (3 out of 5) and 80% (4 out of 5) of the extraintestinal *A. veronii* isolates, respectively. Of the intestinal *A. veronii* isolates examined, only 23% (3 out of 13) had a T3SS and 15% (2 out of 13) had a T6SS. The statistical analysis demonstrated that the more frequent occurrence of the T6SS in extraintestinal *A. veronii* isolates was significant (*p* = 0.0217, Fisher’s exact test).

Taking a similarity level of 80% as a basis, the number of virulence factors present in *A. veronii* was reflected in a division of cluster III into three subclusters, with subcluster 1 (strains 21-AL00085 to 21-AL00083) containing mainly *A. veronii* isolates with ≤8 virulence factors (*A. veronii* basic profile), subcluster 2 (strains 21-AL00084 to 21-AL00141) comprising primarily *A. veronii* isolates with nine to 10 virulence traits (*A. veronii* basic profile +1 to 2 additional factors), and subcluster 3 (strains 21-AL00142 to 21-AL00013) harboring exclusively *A. veronii* isolates with ≥10 virulence traits (*A. veronii* basic profile +2 or more additional factors). While only intestinal *A. veronii* isolates grouped together in subcluster 1, subcluster 2 and subcluster 3 included both intestinal and extraintestinal isolates.

#### 3.5.2 Cluster II: *Aeromonas hydrophila*

Cluster II contained all human clinical *A. hydrophila* isolates investigated in this study, the *A. dhakensis* isolate 21-AL00132, the *A. bestiarum* isolate 21-AL00126, and the *A. salmonicida* isolate 21-AL00096.

All clinical *A. hydrophila* isolates showed virulence patterns that enclosed 13 to 14 virulence traits. They shared the seven basic virulence factors mentioned above as well as type I pili (c), T6SS (c/p), extracellular hemolysin AHH1 (c), heat-stable cytotonic enterotoxin Ast (c), and amonabactin (c).

At a similarity level of 80%, cluster II split into two subclusters (1: strains 21-AL00096 to 21-AL00091, 2: strains 21-AL00116 to 21-AL00132), with 67% of the clinical *A. hydrophila* isolates of each subcluster harboring aerolysin as an additional virulence factor. Further, RtxA was detected in all *A. hydrophila* isolates of subcluster 1, while a partial T3SS was found in all *A. hydrophila* isolates of subcluster 2. In addition to the partially aerolysin-positive T3SS-positive clinical *A. hydrophila* isolates, subcluster 2 of cluster II included the human clinical *A. dhakensis* isolate 21-AL00132, which was aerolysin-positive and differed from the virulence trait profile of *A. hydrophila* strains of cluster II subcluster 2 only by the lack of the heat-stable cytotonic enterotoxin Ast. In subcluster 1 of cluster II, the partially aerolysin-positive RtxA-positive clinical *A. hydrophila* isolates grouped together with the aerolysin-positive *A. bestiarum* isolate 21-AL00126 and the clinical aerolysin-positive *A. salmonicida* isolate 21-AL00096.

#### 3.5.3 Cluster I: *Aeromonas caviae*

Cluster I contained all human clinical *A. caviae* isolates analyzed in this study, the *A. encheleia* isolate 21-AL00125, the *A. salmonicida* isolate 21-AL00032, and three *Aeromonas* sp. isolates that could not be further typed at the species level.

The *A. caviae* isolates exhibited virulence patterns comprising eight to nine virulence traits. The majority of the clinical *A. caviae* isolates (94%) were characterized by the seven basic virulence factors mentioned above and amonabactin (c).

Based on a similarity level of 98%, cluster I showed low diversity with two larger subclusters and a group of few separate strains. In addition to clinical *A. caviae* isolates, subcluster 1 (strains 21-AL00012 to 21-AL00125) contained the human clinical *A. encheleia* isolate (strain 21-AL00125). All subcluster 1 isolates showed eight virulence factors (*A. caviae* basic profile). Subcluster 2 (strains 21-AL00015 to 21-AL00139) included only clinical *A. caviae* isolates. All subcluster 2 isolates were characterized by nine virulence factors [*A. caviae* basic profile + T6SS (p)]. The non-typeable *Aeromonas* sp. isolates possessed similarity to subcluster 2 isolates. In contrast to the cluster II-*A. salmonicida* isolate and -*A. bestiarum* isolate, the heat-stable cytotonic enterotoxin Ast was not detected in the cluster I-*A. salmonicida* isolate.

## 4 Discussion

### 4.1 Species identification, sources of isolates, and clinical manifestation

The aim of the study was to investigate human clinical isolates by whole genome sequencing to identify the predominant *Aeromonas* species in infections in Germany. In addition, the genome sequences were used to identify putative virulence factors present in the pathogenic isolates. These virulence markers could be used to search for reservoirs of human pathogenic strains according to the One Health approach. Bacteria of the genus *Aeromonas* represent an excellent example for the close link between the health of humans, animals and the environment according to the One Health concept, as they are ubiquitously distributed in aquatic environments, in natural soils and many organisms (Lamy et al., 2022).

In total, 52 clinical *Aeromonas* isolates were studied that had been collected in Germany from 2016 to 2019. The isolates were all from human patients with a large variability of disease symptoms. *Aeromonas* bacteria are well known to cause a wide spectrum of illnesses comprising intestinal infections and a variety of extraintestinal infections, e.g., septicemia, wound infection, urinary tract infections, skin and soft tissue infections, as well as pneumonia (Janda and Abbott, 2010; Gonçalves Pessoa et al., 2019).

As our ANI analysis revealed, the majority of isolates belonged to the three species *A. caviae* (17 isolates), *A. hydrophila* (nine isolates), and *A. veronii* biovar *sobria* (18 isolates). These three species have been identified as responsible for the majority of infections in humans (Janda and Abbott, 2010; Pessoa et al., 2022), although frequency of the species may differ according to the region where they were isolated (Pessoa et al., 2022). As this is the first study on clinical *Aeromonas* isolates in Germany, no comparison of data on the occurrence of *Aeromonas* for this region is available. Other *Aeromonas* species associated with human diseases were also identified. One isolate belonged to the species *A. dhakensis*. Strains of this species were previously often mistaken for *A. hydrophila* (Hoel et al., 2019), but they are now recognized as common isolates from infected humans (Gonçalves Pessoa et al., 2019; Fernández-Bravo and Figueras, 2020). One isolate of the species *A. bestiarum* and two *A. salmonicida* isolates were among the patients’ isolates. Three closely related isolates could not be assigned to any described *Aeromonas* species and were termed *Aeromonas* sp.. Finally, one isolate belonged to the hitherto not with human illness associated species *A. encheleia*. However, the isolate was found together with an *A. bestiarum* isolate from a patient with a bursitis, so its putative role in disease is questionable.

The submitted *Aeromonas* isolates were divided into two groups based on the infection type (intestinal vs. extraintestinal infection). All isolates from stool samples were categorized as intestinal, whereas the remaining extraintestinal isolates revealed a high variability of clinical manifestation in the diseased patients (Tables 2, 3). Though only anonymized epidemiological data referring to patient data were available, some conclusions can be drawn. There was no statistically significant difference concerning the gender and the geographical origin (coastal vs. non-coastal regions; Table 4). As *Aeromonas* spp. are inhabitants of aquatic environments, fish and seafood are regarded as a common source of these bacteria (Gonçalves Pessoa et al., 2019). However, in our study there were no indications that *Aeromonas* infections might be higher in coastal regions. Regarding the age of patients, it was clearly observed that the highest isolation rate was in the age group above 60 years. Given the characterization of pathogenic *Aeromonas* strains as opportunistic pathogens, the correlation between age and isolation rates can be expected (Janda and Abbott, 2010; Pessoa et al., 2022).

In our study, the majority of isolates (30 out of 52) originated from stool samples of patients with mainly (gastro-)enteritis. The isolation of clinical *Aeromonas* strains from the intestinal tract has been frequently reported; however, the role of *Aeromonas* spp. as enteropathogens has been controversially debated for many years (Janda and Abbott, 2010). In the last years, new studies have been published that report outbreaks of infections due to the consumption of contaminated drinking water and food (reviewed in Hoel et al., 2019), thus supporting the significance of *Aeromonas* spp. as enteropathogens (Teunis and Figueras, 2016; Pessoa et al., 2022). The above-mentioned intestinal isolates in our study were all from patients with (gastro-)enteritis and no other possible enteropathogenic bacteria had been detected in the stool samples. However, information about the probable source of infection is not available. The common occurrence of the *Aeromonas* species *A. caviae*, *A. veronii*, and *A. hydrophila* in diarrheal patients has been reported worldwide, although the frequency of the species may vary depending on the geographical origin (Pessoa et al., 2022). The species *A. salmonicida* is known as a fish pathogen that grows at lower temperature; however, mesophilic human pathogenic strains have evolved, in which major genetic rearrangements were detected compared to the psychrophilic strains (Fernández-Bravo and Figueras, 2020). Three intestinal isolates that formed a distinct group in the phylogenetic tree (Figure 1) could not be assigned to a recognized *Aeromonas* species. The ANI values obtained through comparison with reference genomes suggest a close relationship to *A. media*/*A. rivipollensis* but the scores are just below the cut-off score suggested for species identification*. A. media* is a species that has been isolated from intestinal and extraintestinal infections (Pessoa et al., 2022).

In a large study on *Aeromonas* strains isolated from patients with gastroenteritis in Australia (Yuwono et al., 2021), a correlation between infection rate and increasing age was reported. Regarding the isolation rate through bacterial cultivation, *Aeromonas* sp. (no species identification) was the third most common bacterial enteropathogen following *Campylobacter* and *Salmonella*. In contrast to the two other enteropathogens, the *Aeromonas* isolation rate was clearly associated with the increase of patient age (Yuwono et al., 2021). In a continuation of this study (Yuwono et al., 2023), qPCR was performed on more than 300,000 stool samples (collected between 2015 and 2019) of patients and it was found that *Aeromonas* spp. were the second most common bacterial pathogens in patients with gastroenteritis after *Campylobacter*. *Aeromonas* enteric infections showed an age-related pattern with three peaks related to age. Young children with an age between 0 and 4 years had an increased infection rate, a small peak was observed for young adults (20–29 years of age) and an increasing infection rate starting with patient’s age from 50 years on. In our study, only three intestinal isolates originated from patients aged up to 10 years, the highest number of isolates stemmed from patients over 60 years of age. Most (gastro-)enteritis isolates (23 out of 28) belonged to the species *A. caviae* and *A. veronii*, while only one out of nine *A. hydrophila* isolates was isolated from stool. The three closely related *Aeromonas* sp. isolates of our study were also from patients with (gastro-)enteritis.

A recent study (Klemm et al., 2024) compared the species diversity and gene contents of *Aeromonas* isolates from feces of young Pakistani children (0–5 years) collected in the years 2009 to 2012. A large-scale genomic analysis was conducted on isolates from children suffering from moderate-to-severe diarrhea and on isolates from a case-matched control group. The analysis showed no clear evidence of differences between the isolates of the two groups in terms of the *Aeromonas* species identified (mainly *A. caviae*, *A. veronii*, and *A. dhakensis*) and the genes associated with their virulence. Interestingly, the complete absence of strains of the species *A. hydrophila* was noted. Aeromonads had previously been identified by GEMS (Global Enteric Multicenter Study) as a significant cause of diarrhea in Pakistani children, taking into account the presence of other pathogens and socioeconomic factors (Qamar et al., 2016). In our study, three of the four isolates from children aged up to 10 years were intestinal isolates (2x *A. caviae* and 1x *A. veronii*). Notably, only one of the nine *A. hydrophila* isolates of our study originated from an intestinal infection.

The 22 isolates from extraintestinal infections originated from a variety of human specimens and reflect the diverse kinds of diseases caused by *Aeromonas* (Tables 2, 3). The astonishingly wide spectrum of diseases associated with *Aeromonas* has been frequently described (Janda and Abbott, 2010; Parker and Shaw, 2011; Gonçalves Pessoa et al., 2019; Fernández-Bravo and Figueras, 2020). Most extraintestinal infections are wound infections, followed by septicemia (Parker and Shaw, 2011; Fernández-Bravo and Figueras, 2020). Specimens from wound infections were also the second most frequent source of the clinical isolates of this study (nine strains), with five isolates belonging to *A. hydrophila*.

Often, *Aeromonas* infections involve more than one type of bacteria in the same clinical sample and are reported as polymicrobial infections. Samples from mixed infections may contain more than one *Aeromonas* isolate (e.g., like *A. bestiarum* and *A. encheleia* in our study) or may contain bacteria of other pathogenic species. Many polymicrobial infections originate from patients with extraintestinal infections (pneumonia, cholangitis cases, surgical site infections, wound infection, bacteremia (Fernández-Bravo and Figueras, 2020)). In a recent study from Australia (Yuwono et al., 2021), in 18% of stool samples *Aeromonas* strains were co-isolated with other human enteric pathogens. As part of the metadata of our study, the submitting laboratories were questioned concerning isolation of other bacteria in the same biological sample; however, co-isolation with other bacteria was reported only for extraintestinal infections.

### 4.2 Genetic relationships of isolates and virulence factor profiles

The genome sequences were used for an alignment including publicly available reference sequences (Figure 1). The three main species (*A. caviae*, *A. hydrophila*, and *A. veronii*) formed distinct genetic clusters. All other isolates were assigned to other species (*A. bestiarum*, *A. dhakensis*, *A. encheleia*, *A. salmonicida*, and *Aeromonas* sp.) and were clearly separated in the phylogenetic tree (Figure 1). To gain more insight into the genetic relationship between the isolates, we performed a core SNP analysis of each species for which several isolates were available (*A. caviae*, *A. veronii*, and *A. hydrophila*). The analysis showed that most isolates of each species are very different from each other (> several 10,000 SNPs). Only two exceptions were found, one pair of *A. hydrophila* isolates differed only by less than 30 SNPs and one pair of *A. veronii* isolates by less than 80 SNPs. From the metadata of these isolates, it is apparent that they were independently isolated (different years of isolation and patient gender).

To determine virulence gene profiles, a second grouping of all clinical isolates of this study was performed by determining if sequences of virulence factors were present, absent, or partially present in the genomes (see Supplementary Table S2 for criteria of partially absent). Virulence factors were grouped into five different functional categories (motility, adhesion, effector delivery systems, exotoxins, nutritional/metabolic factors). The sequences of 288 genes associated with pathogenicity were obtained from the “virulence factor database” (VFDB; http://www.mgc.ac.cn/cgi-bin/VFs/genus.cgi?Genus=Aeromonas) (Table 1).

Sequences of 119 genes coding for components of polar and lateral flagella were used in the screening, as *Aeromonas* strains associated with human disease are motile (and mesophilic), while non-motile, psychrophilic strains growing at lower temperatures are pathogenic for fish (Pund and Theegarten, 2008; Janda and Abbott, 2010; Pessoa et al., 2022). Motility of bacteria involves movement in liquid environments by polar flagella and movement on solid surfaces by lateral flagella (Gonçalves Pessoa et al., 2019). Motility is associated with virulence by contributing to adhesion and biofilm formation. Genes coding for polar and/or lateral flagella are organized in several gene clusters (Tomás, 2012) and were detected in all investigated isolates (Supplementary Table S2), although the number of detected genes varied considerably. Recently, two genes, *maf2* and *lafT*, that encode proteins associated with components of the polar and lateral flagella function and biogenesis were identified to be correlated with moderate-to-severe diarrhea in Pakistani children, though this correlation needs further investigation (Klemm et al., 2024). Both genes were present in some isolates of our study. Six *A. hydrophila* isolates and three isolates from other species harbored the *maf2* gene, and 10 *A. veronii* isolates, six *A. hydrophila* isolates, three *A. caviae* isolates, and four isolates from other species possessed the *lafT* gene (see Supplementary Table S2). The two genes were present in isolates from extraintestinal and intestinal infections.

The results of the presence/absence of genes associated with motility were not included in the virulence profile, as the role of individual genes has yet to be determined. It has been demonstrated that mutations in some flagella genes (Qin et al., 2016) affected motility and reduced adhesion and virulence in the infection process.

For the determination of virulence factor profiles, we analyzed the presence/partial presence/absence data of 16 recognized virulence factors (single genes as well as complex structures like secretion systems) assigned to four categories (adhesion, effector delivery systems, exotoxins, nutritional/metabolic factors) (Figure 3). In total, 31 different virulence factor profiles were obtained. The number of potential virulence factors described for human pathogenic strains suggests that *Aeromonas* infections are multifactorial, but the role of many factors is not yet clear (Hoel et al., 2019; Fernández-Bravo and Figueras, 2020).

Three different clusters regarding virulence factor profiles were obtained from the set of the 16 virulence factors based on a similarity level of 55%. Seven virulence factors were present in all isolates (the adherence factors MSHA pili and Tap type IV pili, the effector delivery system T2SS, the exotoxins hemolysin HlyA, hemolysin III, and thermostable hemolysin, and the heme uptake system).

Cluster III comprised only the *A. veronii* biovar *sobria* isolates (18 isolates) of our study. The cluster could be divided into three subclusters. We tried to identify if the presence of virulence factor genes could be associated with a specific type of infection (intestinal vs. extraintestinal *Aeromonas* infections). However, only for five virulence factors significant differences regarding their occurrence were found. Genes of the T6SS were significantly more frequently present in extraintestinal *A. veronii* isolates (4 out of 5; 80%) than in intestinal *A. veronii* isolates (15%). T6SSs are able to inject effector proteins into the cytosol of target cells and are likely to play a role against competing bacteria in the respective environment, which can be bacteria in a polymicrobial infection (Fernández-Bravo and Figueras, 2020). Interestingly, the *A. veronii* isolates did not harbor several exotoxin genes (*rtxA*, *ast*, hemolysin AHH1-coding gene) and did not possess genes encoding the synthesis of the amonabactin siderophore. This siderophore is a specific siderophore produced by some *Aeromonas* species (Tomás, 2012).

In comparison, cluster III strains (*A. veronii*) possess fewer virulence factor genes than the cluster II strains, of which *A. hydrophila* is the dominant species in our study. In other studies (Talagrand-Reboul et al., 2020), an uneven distribution of virulence genes between different phylogroups was observed, with *A. hydrophila/A. dhakensis* carrying more virulence-associated genes than other groups. In an investigation comparing strains of several *Aeromonas* species by examining the presence of a set of seven virulence factor genes by PCR, it was found that *A. hydrophila* strains possessed more of these genes than strains of the species *A. veronii* and *A. caviae* (Silva et al., 2017). We noticed also that *A. caviae*, the dominant species of cluster I, had fewer virulence factor genes than cluster II strains. The authors hypothesized that the higher content of virulence factor genes might indicate a higher virulence potential of the species *A. hydrophila*. However, they also pointed out that the infection process of the two other species might require different pathogenicity mechanisms and might explain differences in gene contents (Silva et al., 2017). In a study analyzing the presence of another set of virulence factor genes in *Aeromonas* strains from diseased ornamental fish, *A. hydrophila* strains also harbored more of these genes than strains of the species *A. caviae* and *A. veronii* (de Oliveira et al., 2024).

Cluster II strains consisted of nine *A. hydrophila* isolates and one isolate each of the species *A. dhakensis*, *A. bestiarum*, and *A. salmonicida*. While *A. dhakensis* is more closely related to *A. hydrophila* (Figure 1), the other two isolates are phylogenetically distinct. Interestingly, most cluster II strains were isolated from extraintestinal infections (11 strains out of 12) with only one *A. hydrophila* isolate from an intestinal infection. However, *A. hydrophila* has been often recovered from patients with diarrhea (Fernández-Bravo and Figueras, 2020), so our isolate collection may be atypical in this context. Nevertheless, it is worth mentioning here that in the study on young Pakistani children suffering from diarrhea, not a single *A. hydrophila* strain was detected (Klemm et al., 2024).

Two subgroups of cluster II strains defined at a similarity level of 80% differ regarding the presence of genes encoding a T3SS and RtxA. In one subcluster, T3SS genes encoding structural components of the secretion system were detected, while based on the criteria (Supplementary Table S2) only one gene for a secreted effector (gene *ABH06556*) was detected in some isolates. The secretion apparatus of T3SSs is homologous to the flagellum and can be detected with bioinformatic searches, whereas the T3SS effector genes are less conserved and often possess uncharacterized domains which requires more bioinformatic effort (Rangel et al., 2019). This could explain why only one effector gene (out of six examined; Table 1) was identified in few isolates. The second subcluster did not contain T3SS genes but a gene cluster coding for repeat-in-toxin RtxA and additional proteins involved in regulation and activation of the RtxA protein (genes *rtxB-E* and *H*). The RtxA proteins are large proteins with variable effector domains that bind to the cell plasma membrane of host cells. After translocation into the host cell, the multifunctional protein is processed and its effector domains are released into the cytosol (Kim, 2018). It is notable that in cluster II the two subgroups differ by possession of either T3SS or RtxA.

Strains of cluster I are mostly *A. caviae* isolates that do not possess an aerolysin gene. Aerolysin and related cytotoxic enterotoxins are well-known pore-forming toxins and were described many years ago in other *Aeromonas* species (e.g., *A. hydrophila*, *A. veronii*, and *A. dhakensis*), but can be found in some *A. caviae* strains (Hoel et al., 2019). The three *Aeromonas* isolates that were not assigned to a recognized species belonged to cluster I.

### 4.3 *Aeromonas* and the One Health agenda

The ubiquitous presence of *Aeromonas* in diverse environments as well as their association with many different organisms characterize these bacteria as important microorganisms within the One Health agenda (Lamy et al., 2022). The One Health approach recognizes that global health risks, like, e.g., zoonotic diseases or antimicrobial resistance, are interconnected and need an integrated, unifying collaboration of public health institutions to tackle threats to health and ecosystems (World Health Organization; see https://www.who.int/europe/initiatives/one-health). Our study aims to characterize human pathogenic *Aeromonas* isolates and to use this information to identify their reservoirs in animals and food as well as in the environment.

The clustering of isolates based on virulence factor profiles resulted in a grouping that closely resembles the clustering of the phylogenetic tree (Figure 3). This suggests that the profile of the investigated 16 virulence factors may be specific for each species. When searching for reservoirs of human pathogenic strains, these factors could be used as markers for the screening. However, it must be taken into account that *Aeromonas* infections are multifactorial and several more putative virulence factors may contribute to the various types of infection (Hoel et al., 2019; Fernández-Bravo and Figueras, 2020). It has yet to be determined how many of the various exoenzymes of *Aeromonas* spp. (e.g., lipases, proteases, amylases, nucleases etc.) secreted under changing growth conditions in different environments contribute to the pathogenicity of strains in human infections (Tomás, 2012; Gonçalves Pessoa et al., 2019). Additionally, structural components (e.g., lipopolysaccharide, capsules, S-layer, flagella) must be considered for the virulence of pathogenic strains.

*Aeromonas* infections are known to increase in summer, when temperatures in the environment are elevated and the frequency of bacteria in aquatic environments is higher compared to colder seasons (Parker and Shaw, 2011; Yuwono et al., 2021). The seasonality of infections is well documented for intestinal infections but has also been noted for extraintestinal infections, though the number of these infections is lower (Janda and Abbott, 2010). Therefore, in view of rising mean temperatures attributable to climate change human exposure to *Aeromonas* is expected to increase along with the number of infections (Klemm et al., 2024). From a public health perspective it should be considered to change *Aeromonas* infections from a non-notifiable to a notifiable status, as this would improve the tracing of sources or reservoirs of these bacteria also in the environment. In this context, it is encouraging that despite the great diversity between most isolates on species level as detected by the SNP analysis, two pairs of isolates were identified (one pair in the species *A. hydrophila* and one pair in the species *A. veronii*) that were closely genetically related but clearly originated from different epidemiological events. With a reporting system in place, it should be possible to identify the primary source of those infections.

## 5 Conclusion

The study was undertaken to characterize for the first time clinical *Aeromonas* isolates from Germany. The isolates originated from patients with intestinal infections and extraintestinal infections with a variety of disease symptoms. Most of the German isolates belonged to the three species *A. veronii*, *A. caviae*, and *A. hydrophila* that are worldwide responsible for the majority of human infections attributed to this genus. *Aeromonas* bacteria are present in the environment, in food, in animals, and in humans, which according to the One Health concept, demands integrative and comprehensive research to identify possible reservoirs of these bacteria. Despite regular cases of illness, it is still unclear from which One Health sources in Germany the human infections with bacteria of the *Aeromonas* group originate. The knowledge gap on pathogenic *Aeromonas* is also caused by the lack of notification/reporting for these bacteria. Through whole genome sequencing of patient isolates, data were obtained on human pathogenic *Aeromonas* bacteria in Germany. With this knowledge, work will continue to develop and establish rapid and reliable diagnostic techniques to identify pathogenic *Aeromonas* bacteria in One Health sources in Germany.

## Data Availability

The datasets presented in this study can be found in online repositories. The names of the repository/repositories and accession number(s) can be found in the article/Supplementary Table S1.

## Footnotes

^1^ https://www.ezbiocloud.net/tools/ani

^2^ https://github.com/tseemann/snippy

^3^ https://github.com/tseemann/snp-dists

^4^ http://www.mgc.ac.cn/VFs/

^5^ https://www.ncbi.nlm.nih.gov/sra

^6^ https://www.ncbi.nlm.nih.gov/refseq/

## German *Aeromonas* Study Group

Veronika Balau, IMD Labor Greifswald, MVZ Labor Greifswald GmbH, Greifswald, Germany; Cornelia Göllner, German Federal Institute for Risk Assessment, Berlin, Germany; Jana Kuhn, IMD Labor Greifswald, MVZ Labor Greifswald GmbH, Greifswald, Germany; Claudia Metelmann, IMD Labor Greifswald, MVZ Labor Greifswald GmbH, Greifswald, Germany; Jonas Nekat, German Federal Institute for Risk Assessment, Berlin, Germany; Arthur Pranada, MVZ Dr. Eberhard & Partner Dortmund GbR, Dortmund, Germany; Denise Rose, IMD Labor Oderland GmbH, MVZ Ärztliches Labor Dr. Frank Berthold und Kollegen, Frankfurt (Oder), Germany; Rabea Schikarski, MVZ Dr. Eberhard & Partner Dortmund GbR, Dortmund, Germany and MVZ Medlabwahl GmbH, Lüdenscheid, Germany; Ilona Schwede, IMD Labor Oderland GmbH, MVZ Ärztliches Labor Dr. Frank Berthold und Kollegen, Frankfurt (Oder), Germany.
