# Supplementary material for: Emerging Aeromonas spp. infections in Europe: characterization of human clinical isolates from German patients
Source: Front Microbiol. 2024 Dec 18;15:1498180. doi: 10.3389/fmicb.2024.1498180 (PMC11688387; doi:10.3389/fmicb.2024.1498180)
Supplement: Supplementary file 3 [file Table_3.pdf]

Table S3. Whole genome sequence (WGS)-based species results of *Aeromonas* spp. strains (*n* = 52) from German patients (*n* = 51). Species pre-identification was performed using the Bacterial and Viral Bioinformatics Resource Center's (BV-BRC) Similar Genome Finder (SGF) tool (https://www.bv-brc.org/app/GenomeDistance; v3.28.9). In SGF analyses, fasta sequences of the *Aeromonas* spp. strains from German patients (21-ALXXXXX identifier) as well as all publicly available bacterial and archaeal genomes from the BV-BRC database were included. Starting with the first (best) BV-BRC SGF-match, up to five SGF-based species results were verified using the EzBioCloud's Average Nucleotide Identity (ANI) calculator tool (https://www.ezbiocloud.net/tools/ani). In ANI analyses, fasta sequences of the *Aeromonas* spp. strains from German patients (21-ALXXXXX identifier) and *Aeromonas* sp. type strain genomes (reference genomes) from the National Center for Biotechnology Information's (NCBI) Reference Sequence (RefSeq) database served as input files. *Aeromonas* strains that could not be reliably identified at the species level (OrthoANIu values ≤ 95%), were referred to as "*Aeromonas* sp.".

**Strain**	**BV-BRC Similar Genome Finder**					**EzBioCloud's ANI calculator**		**WGS-based species result**
	**Genome name (species, strain)**	**GenBank accessions**	**Distance**	**P value**	**K-mer counts**	**Reference genome (species, strain, NCBI RefSeq accession number)**	**OrthoANIu value (%)**	
**Intestinal (*n* = 30)**								
**21-AL00012**	Aeromonas caviae Aer268	RQJY00000000	0.0132573	0	609/1000	Aeromonas caviae_NCTC12244_NZ_LS483441.1.fasta	97.81	*Aeromonas caviae*
**21-AL00013**	Aeromonas veronii AK247	NKXD00000000	0.0265635	0	401/1000	Aeromonas veronii_CECT 4257_NZ_CDDK00000000.1.fasta	96.50	*Aeromonas veronii*
**21-AL00015**	Aeromonas caviae BVH84	JAAALW000000000	0.0113529	0	650/1000	Aeromonas caviae_NCTC12244_NZ_LS483441.1.fasta	97.91	*Aeromonas caviae*
**21-AL00021**	Aeromonas caviae CN17A0191	JAEHIL000000000	0.015616	0	563/1000	Aeromonas caviae_NCTC12244_NZ_LS483441.1.fasta	97.78	*Aeromonas caviae*
**21-AL00032**	Aeromonas salmonicida Colony462	CP080045	0.0230043	0	446/1000	Aeromonas salmonicida_NCTC12959_NZ_UAPT00000000.1.fasta	96.96	*Aeromonas salmonicida*
**21-AL00040**	Aeromonas media BAQ071013-115	NKWY00000000	0.0168391	0	541/1000	Aeromonas media_CECT 4232_NZ_CDBZ00000000.1.fasta	93.81	*Aeromonas* sp.
	Aeromonas sp. 3925	JAAROG000000000	0.0211548	0	472/1000	Aeromonas rivipollensis_KN-Mc-11N1_NZ_CP027856.1.fasta	94.53	
	Aeromonas media strain CN17A0010	JAEHIH000000000	0.0212234	0	471/1000			
	Aeromonas media strain Colony414	CP070623	0.0368186	0	300/1000			
	Aeromonas rivipollensis strain KN-Mc-11N1	CP027856	0.0393656	0	280/1000			
	Aeromonas media strain BAQ071013-132	NKWW00000000	0.0400373	0	275/1000			
**21-AL00052**	Aeromonas veronii A7	JAIEYB000000000	0.025894	0	409/1000	Aeromonas veronii_CECT 4257_NZ_CDDK00000000.1.fasta	96.42	*Aeromonas veronii*
**21-AL00066**	Aeromonas sp. ASNIH7	CP026226,CP026227	0.00997914	0	682/1000	Aeromonas hydrophila_ATCC 7966_NC_008570.1.fasta	86.63	*Aeromonas caviae*
	Aeromonas hydrophila strain ZJ66-1	UETG00000000	0.0100207	0	681/1000	Aeromonas caviae_NCTC12244_NZ_LS483441.1.fasta	97.81	
	Aeromonas caviae strain ZJ66-1	NXBP00000000	0.0100207	0	681/1000			
	Aeromonas caviae strain WP8-S17-ESBL-03	AP022214	0.0108269	0	662/1000			
	Aeromonas caviae strain BVH84	JAAALW000000000	0.0118017	0	640/1000			
	Aeromonas caviae strain 1507-17068	CP047982	0.0118017	0	640/1000			
**21-AL00070**	Aeromonas media BAQ071013-115	NKWY00000000	0.0170111	0	538/1000	Aeromonas media_CECT 4232_NZ_CDBZ00000000.1.fasta	93.83	*Aeromonas* sp.
	Aeromonas sp. 3925	JAAROG000000000	0.0196275	0	495/1000			
	Aeromonas media strain CN17A0010	JAEHIH000000000	0.0209501	0	475/1000			
	Aeromonas media strain Colony414	CP070623	0.0357395	0	309/1000			
	Aeromonas media strain BAQ071013-132	NKWW00000000	0.0405854	0	271/1000			
	Aeromonas media strain CIP 103208	JAGDES000000000	0.040724	0	270/1000			
**21-AL00072**	Aeromonas caviae D	VZQB00000000	0.0149759	0	575/1000	Aeromonas caviae_NCTC12244_NZ_LS483441.1.fasta	97.89	*Aeromonas caviae*
**21-AL00078**	Aeromonas caviae Aer593	RQJZ00000000	0.014871	0	577/1000	Aeromonas caviae_NCTC12244_NZ_LS483441.1.fasta	97.86	*Aeromonas caviae*
**21-AL00079**	Aeromonas sp. 3925	JAAROG000000000	0.00734051	0	750/1000	Aeromonas media_CECT 4232_NZ_CDBZ00000000.1.fasta	93.87	*Aeromonas* sp.
	Aeromonas media strain BAQ071013-115	NKWY00000000	0.0180709	0	520/1000	Aeromonas rivipollensis_KN-Mc-11N1_NZ_CP027856.1.fasta	94.55	
	Aeromonas media strain CN17A0010	JAEHIH000000000	0.0221335	0	458/1000			
	Aeromonas media strain Colony414	CP070623	0.0365754	0	302/1000			
	Aeromonas rivipollensis strain KN-Mc-11N1	CP027856	0.0414262	0	265/1000			
	Aeromonas media strain BAQ071013-132	NKWW00000000	0.0415686	0	264/1000			
**21-AL00081**	Aeromonas veronii ADV102	NKXH00000000	0.0127293	0	620/1000	Aeromonas veronii_CECT 4257_NZ_CDDK00000000.1.fasta	96.46	*Aeromonas veronii*
**21-AL00083**	Aeromonas veronii CN17A0059	JAEHHQ000000000	0.0272504	0	393/1000	Aeromonas veronii_CECT 4257_NZ_CDDK00000000.1.fasta	96.56	*Aeromonas veronii*

Table continued								
**Strain**	**BV-BRC Similar Genome Finder**					**EzBioCloud's ANI calculator**		**WGS-based species result**
	**Genome name (species, strain)**	**GenBank accessions**	**Distance**	**P value**	**K-mer counts**	**Reference genome (species, strain, NCBI RefSeq accession number)**	**OrthoANIu value (%)**	
**21-AL00084**	Aeromonas veronii CN17A0054	JAEHHT000000000	0.0177716	0	525/1000	Aeromonas veronii_CECT 4257_NZ_CDDK00000000.1.fasta	96.53	*Aeromonas veronii*
**21-AL00085**	Aeromonas veronii 126-14	PPTE00000000	0.00723198	0	753/1000	Aeromonas veronii_CECT 4257_NZ_CDDK00000000.1.fasta	96.44	*Aeromonas veronii*
**21-AL00105**	Aeromonas caviae Colony23	CP081293	0.0170111	0	538/1000	Aeromonas caviae_NCTC12244_NZ_LS483441.1.fasta	97.85	*Aeromonas caviae*
**21-AL00110**	Aeromonas caviae BVH98	JAAALV000000000	0.00126265	0	949/1000	Aeromonas caviae_NCTC12244_NZ_LS483441.1.fasta	97.91	*Aeromonas caviae*
**21-AL00111**	Aeromonas caviae KAM463	BQVH00000000	0.017653	0	527/1000	Aeromonas caviae_NCTC12244_NZ_LS483441.1.fasta	97.74	*Aeromonas caviae*
**21-AL00113**	Aeromonas caviae KAM334	BPNB00000000	0.0166683	0	544/1000	Aeromonas caviae_NCTC12244_NZ_LS483441.1.fasta	97.76	*Aeromonas caviae*
**21-AL00115**	Aeromonas hydrophila KN-Mc-1R2	CP027804	0.0260597	0	407/1000	Aeromonas hydrophila_ATCC 7966_NC_008570.1.fasta	96.80	*Aeromonas hydrophila*
**21-AL00118**	Aeromonas caviae BVH84	JAAALW000000000	0.010441	0	671/1000	Aeromonas caviae_NCTC12244_NZ_LS483441.1.fasta	97.83	*Aeromonas caviae*
**21-AL00128**	Aeromonas veronii 126-14	PPTE00000000	0.0102298	0	676/1000	Aeromonas veronii_CECT 4257_NZ_CDDK00000000.1.fasta	96.32	*Aeromonas veronii*
**21-AL00131**	Aeromonas veronii A134	RSFC00000000	0.0273375	0	392/1000	Aeromonas veronii_CECT 4257_NZ_CDDK00000000.1.fasta	96.55	*Aeromonas veronii*
**21-AL00133**	Aeromonas veronii 126-14	PPTE00000000	0.00708822	0	757/1000	Aeromonas veronii_CECT 4257_NZ_CDDK00000000.1.fasta	96.49	*Aeromonas veronii*
**21-AL00134**	Aeromonas veronii FDAARGOS_632	CP044060,CP044061	0.028588	0	378/1000	Aeromonas veronii_CECT 4257_NZ_CDDK00000000.1.fasta	96.52	*Aeromonas veronii*
**21-AL00136**	Aeromonas veronii A20-8	JAIEYJ000000000	0.0255655	0	413/1000	Aeromonas veronii_CECT 4257_NZ_CDDK00000000.1.fasta	96.56	*Aeromonas veronii*
**21-AL00139**	Aeromonas caviae Colony274	CP081290	0.0116661	0	643/1000	Aeromonas caviae_NCTC12244_NZ_LS483441.1.fasta	97.83	*Aeromonas caviae*
**21-AL00142**	Aeromonas veronii A21-13	JAIEYM000000000	0.0258115	0	410/1000	Aeromonas veronii_CECT 4257_NZ_CDDK00000000.1.fasta	96.38	*Aeromonas veronii*
**21-AL00143**	Aeromonas veronii CN17A0036	JAEHHZ000000000	0.0129198	0	616/1000	Aeromonas veronii_CECT 4257_NZ_CDDK00000000.1.fasta	96.36	*Aeromonas veronii*
**Extraintestinal (*n* = 22)**								
**21-AL00018**	Aeromonas caviae CECT 838	JAGDEN000000000	0.0152936	0	569/1000	Aeromonas caviae_NCTC12244_NZ_LS483441.1.fasta	98.28	*Aeromonas caviae*
**21-AL00065**	Aeromonas caviae Sch29	CAAKNG000000000	0.0155619	0	564/1000	Aeromonas caviae_NCTC12244_NZ_LS483441.1.fasta	97.73	*Aeromonas caviae*
**21-AL00068**	Aeromonas veronii CN17A0067	JAEHHO000000000	0.0276889	0	388/1000	Aeromonas veronii_CECT 4257_NZ_CDDK00000000.1.fasta	96.40	*Aeromonas veronii*
**21-AL00075**	Aeromonas veronii CN17A0013	JAEHIF000000000	0.0132573	0	609/1000	Aeromonas veronii_CECT 4257_NZ_CDDK00000000.1.fasta	96.35	*Aeromonas veronii*
**21-AL00076**	Aeromonas hydrophila AH10	CP011100	0.00454897	0	833/1000	Aeromonas hydrophila_ATCC 7966_NC_008570.1.fasta	97.01	*Aeromonas hydrophila*
**21-AL00080**	Aeromonas veronii BVH46	NKWS00000000	0.0275126	0	390/1000	Aeromonas veronii_CECT 4257_NZ_CDDK00000000.1.fasta	96.46	*Aeromonas veronii*
**21-AL00082**	Aeromonas hydrophila KAM461	BQVF00000000	0.0243695	0	428/1000	Aeromonas hydrophila_ATCC 7966_NC_008570.1.fasta	96.99	*Aeromonas hydrophila*
**21-AL00086**	Aeromonas caviae Aer593	RQJZ00000000	0.0177122	0	526/1000	Aeromonas caviae_NCTC12244_NZ_LS483441.1.fasta	97.83	*Aeromonas caviae*
**21-AL00088**	Aeromonas hydrophila NF1	JDWB00000000	0.00662843	0	770/1000	Aeromonas hydrophila_ATCC 7966_NC_008570.1.fasta	96.84	*Aeromonas hydrophila*
**21-AL00091**	Aeromonas hydrophila Aer_Brac14A	CP045502	0.0237537	0	436/1000	Aeromonas hydrophila_ATCC 7966_NC_008570.1.fasta	97.11	*Aeromonas hydrophila*
**21-AL00095**	Aeromonas caviae CN17A0028	JAEHIC000000000	0.0185579	0	512/1000	Aeromonas caviae_NCTC12244_NZ_LS483441.1.fasta	97.64	*Aeromonas caviae*
**21-AL00096**	Aeromonas salmonicida Colony418	CP080041	0.0197565	0	493/1000	Aeromonas salmonicida_NCTC12959_NZ_UAPT00000000.1.fasta	97.41	*Aeromonas salmonicida*
**21-AL00103**	Aeromonas caviae CN17A0038	JAEHHY000000000	0.0150285	0	574/1000	Aeromonas caviae_NCTC12244_NZ_LS483441.1.fasta	97.98	*Aeromonas caviae*
**21-AL00116**	Aeromonas hydrophila NCTC8049	UFSL00000000	0.0254028	0	415/1000	Aeromonas hydrophila_ATCC 7966_NC_008570.1.fasta	96.85	*Aeromonas hydrophila*
**21-AL00124**	Aeromonas veronii A20-8	JAIEYJ000000000	0.0286795	0	377/1000	Aeromonas veronii_CECT 4257_NZ_CDDK00000000.1.fasta	96.48	*Aeromonas veronii*

Table continued

Strain	BV-BRC Similar Genome Finder					EzBioCloud's ANI calculator		WGS-based species result
	**Genome name (species, strain)**	**GenBank accessions**	**Distance**	**P value**	**K-mer counts**	**Reference genome (species, strain, NCBI RefSeq accession number)**	**OrthoANIu value (%)**	
**21-AL00125**[a]	Aeromonas sp. sif2433	JAHUXD000000000	0.0168391	0	541/1000	Aeromonas encheleia_CECT 4342_NZ_CDDI00000000.1.fasta	97.62	*Aeromonas encheleia*
	Aeromonas encheleia CECT 4342	CDDI00000000	0.0168963	0	540/1000			
	Aeromonas encheleia strain NCTC12917	LR134376	0.0168963	0	540/1000			
	Aeromonas sp. sif2416	JAHUXC000000000	0.0174761	0	530/1000			
	Aeromonas encheleia H4-C21	CP093843	0.0182524	0	517/1000			
	Aeromonas sp. sia0103	JAHUWA000000000	0.0182524	0	517/1000			
	Aeromonas sp. AE235	JRGL00000000	0.0686914	0	134/1000			
	Aeromonas sp. MR16	JAKZFK000000000	0.0702918	0	129/1000			
	Aeromonas sp. MR16	JAKZFK000000000	0.0702918	0	129/1000			
	Aeromonas sp. 3925	JAAROG000000000	0.0786628	0	106/1000			
	Aeromonas media strain CN17A0010	JAEHIH000000000	0.0807479	0	101/1000			
	Aeromonas rivipollensis strain KN-Mc-11N1	CP027856	0.0811785	0	100/1000			
**21-AL00126**[a]	Aeromonas sp. MR19	JAKZFL000000000	0.0202797	0	485/1000	Aeromonas bestiarum_CECT 4227_NZ_CDDA00000000.1.fasta	97.31	*Aeromonas bestiarum*
	Aeromonas sp. MR19	JAKZFL000000000	0.0202797	0	485/1000			
	Aeromonas sp. MR7	JAKZFJ000000000	0.0219205	0	461/1000			
	Aeromonas sp. MR7	JAKZFJ000000000	0.0219205	0	461/1000			
	Aeromonas sp. CA23	CP023818	0.0219913	0	460/1000			
	Aeromonas bestiarum strain GA97-22	PPUX00000000	0.0220623	0	459/1000			
	Aeromonas bestiarum CECT 4227	CDDA00000000	0.0226378	0	451/1000			
	Aeromonas salmonicida CBA100	JPWL00000000	0.0227838	0	449/1000			
	Aeromonas piscicola strain AH-3	LYXO00000000	0.0414262	0	265/1000			
	Aeromonas piscicola LMG 24783	CDBL00000000	0.0440934	0	247/1000			
**21-AL00130**	Aeromonas hydrophila KAM461	BQVF00000000	0.0243695	0	428/1000	Aeromonas hydrophila_ATCC 7966_NC_008570.1.fasta	96.99	*Aeromonas hydrophila*
**21-AL00132**	Aeromonas dhakensis CIP 107500	CDBH00000000	0.0201478	0	487/1000	Aeromonas dhakensis_CIP 107500_NZ_CDBH00000000.1.fasta	97.61	*Aeromonas dhakensis*
**21-AL00137**	Aeromonas hydrophila NCTC8049	UFSL00000000	0.0233014	0	442/1000	Aeromonas hydrophila_ATCC 7966_NC_008570.1.fasta	97.10	*Aeromonas hydrophila*
**21-AL00140**	Aeromonas sp. 1805	CP038515	0.026143	0	406/1000	Aeromonas hydrophila_ATCC 7966_NC_008570.1.fasta	97.04	*Aeromonas hydrophila*
	Aeromonas hydrophila strain CN17A0062	JAEHHP000000000	0.0263945	0	403/1000			
	Aeromonas hydrophila strain CN17A0055	JAEHHS000000000	0.0263945	0	403/1000			
	Aeromonas hydrophila strain OnP22	WOCC00000000	0.026819	0	398/1000			
	Aeromonas hydrophila strain Aer_LaG34	CP046604	0.0269047	0	397/1000			
	Aeromonas hydrophila strain Aer_OnP4.2	CP046870	0.0269047	0	397/1000			
**21-AL00141**	Aeromonas veronii BVH37	NKWU00000000	0.0200822	0	488/1000	Aeromonas veronii_CECT 4257_NZ_CDDK00000000.1.fasta	96.47	*Aeromonas veronii*

[a] Isolates from the same patient.
