# Supplementary material for: Emerging Aeromonas spp. infections in Europe: characterization of human clinical isolates from German patients
Source: Front Microbiol. 2024 Dec 18;15:1498180. doi: 10.3389/fmicb.2024.1498180 (PMC11688387; doi:10.3389/fmicb.2024.1498180)
Supplement: Supplementary file 5 [file Table_5.pdf]

Table S5. Presence of virulence determinants in *Aeromonas* spp. isolates from German patients based on WGS data.

Virulence determinant[a]		Group of *Aeromonas* spp. isolates[d]									
		Total	Geographical origin			Type of infection		Species			
		C - G (*n* = 52)	cr (*n* = 12)	ncr (*n* = 37)	Unknown (*n* = 3)	int (*n* = 30)	ext (*n* = 22)	*Ac* (*n* = 17)	*Ah* (*n* = 9)	*Av* (*n* = 18)	Others (*n* = 8)
**Adherence**											
**Flp type IV pili**	n_t (p_t)*	9 (17%)	1 (8%)	7 (19%)	1 (33%)	6 (20%)	3 (14%)	0 (0%)	0 (0%)	6 (33%)	3 (38%)
**MSHA type IV pili**	n_t (p_t)	51 (98%)	11 (92%)	37 (100%)	3 (100%)	29 (97%)	22 (100%)	16 (94%)	9 (100%)	18 (100%)	8 (100%)
	n_c (p_c)	26 (50%)	3 (25%)	21 (57%)	2 (67%)	12 (40%)	14 (64%)	0 (0%)	9 (100%)	15 (83%)	2 (25%)
	n_p (p_p)	25 (48%)	8 (67%)	16 (43%)	1 (33%)	17 (57%)	8 (36%)	16 (94%)	0 (0%)	3 (17%)	6 (75%)
**Tap type IV pili**	n_t (p_t)	52 (100%)	12 (100%)	37 (100%)	3 (100%)	30 (100%)	22 (100%)	17 (100%)	9 (100%)	18 (100%)	8 (100%)
	n_c (p_c)	20 (38%)	4 (33%)	14 (38%)	2 (67%)	9 (30%)	11 (50%)	0 (0%)	8 (89%)	9 (50%)	3 (37.5%)
	n_p (p_p)	32 (62%)	8 (67%)	23 (62%)	1 (33%)	21 (70%)	11 (50%)	17 (100%)	1 (11%)	9 (50%)	5 (62.5%)
**Type I pili**	n_t (p_t)	17 (33%)	4 (33%)	11 (30%)	2 (67%)	6 (20%)	11 (50%)	1 (6%)	9 (100%)	3 (17%)	4 (50%)
	n_c (p_c)	16 (31%)	4 (33%)	10 (27%)	2 (67%)	5 (17%)	11 (50%)	1 (6%)	9 (100%)	3 (17%)	3 (37.5%)
	n_p (p_p)	1 (2%)	0 (0%)	1 (3%)	0 (0%)	1 (3%)	0 (0%)	0 (0%)	0 (0%)	0 (0%)	1 (12.5%)
**Effector delivery systems**											
**Exe T2SS**	n_t (p_t)*	52 (100%)	12 (100%)	37 (100%)	3 (100%)	30 (100%)	22 (100%)	17 (100%)	9 (100%)	18 (100%)	8 (100%)
**T3SS[b]**	n_t (p_t)	13 (25%)	1 (8%)	12 (32%)	0 (0%)	4 (13%)	9 (41%)	0 (0%)	6 (67%)	6 (33%)	1 (13%)
	n_c (p_c)	1 (2%)	0 (0%)	1 (2.7%)	0 (0%)	0 (0%)	1 (5%)	0 (0%)	0 (0%)	1 (5.6%)	0 (0%)
	n_p (p_p)	12 (23%)	1 (8%)	11 (29.7%)	0 (0%)	4 (13%)	8 (36%)	0 (0%)	6 (67%)	5 (27.8%)	1 (13%)
**T6SS[b]**	n_t (p_t)	27 (52%)	6 (50%)	18 (49%)	3 (100%)	10 (33%)	17 (77%)	6 (35%)	9 (100%)	6 (33%)	6 (75%)
	n_c (p_c)	10 (19%)	1 (8%)	8 (22%)	1 (33%)	1 (3%)	9 (41%)	0 (0%)	7 (78%)	0 (0%)	3 (37.5%)
	n_p (p_p)	17 (33%)	5 (42%)	10 (27%)	2 (67%)	9 (30%)	8 (36%)	6 (35%)	2 (22%)	6 (33%)	3 (37.5%)
**Exotoxins**											
**Aerolysin[c]**	n_t (p_t)*	30 (58%)	3 (25%)	25 (68%)	2 (67%)	16 (53%)	14 (64%)	0 (0%)	6 (67%)	18 (100%)	6 (75%)
	n_{t1} (p_{t1})*	18 (35%)	2 (17%)	14 (38%)	2 (67%)	9 (30%)	9 (41%)	0 (0%)	6 (67%)	9 (50%)	3 (37.5%)
	n_{t2} (p_{t2})*	12 (23%)	1 (8%)	11 (30%)	0 (0%)	7 (23%)	5 (23%)	0 (0%)	0 (0%)	9 (50%)	3 (37.5%)
**Extracellular hemolysin AHH1**	n_t (p_t)*	13 (25%)	2 (17%)	10 (27%)	1 (33%)	2 (7%)	11 (50%)	0 (0%)	9 (100%)	0 (0%)	4 (50%)
**Heat-stable cytotonic enterotoxin, Ast**	n_t (p_t)*	11 (21%)	2 (17%)	8 (22%)	1 (33%)	1 (3%)	10 (45%)	0 (0%)	9 (100%)	0 (0%)	2 (25%)
**Hemolysin HlyA**	n_t (p_t)*	52 (100%)	12 (100%)	37 (100%)	3 (100%)	30 (100%)	22 (100%)	17 (100%)	9 (100%)	18 (100%)	8 (100%)
**Hemolysin III**	n_t (p_t)*	52 (100%)	12 (100%)	37 (100%)	3 (100%)	30 (100%)	22 (100%)	17 (100%)	9 (100%)	18 (100%)	8 (100%)
**RtxA**	n_t (p_t)	6 (12%)	1 (8%)	4 (11%)	1 (33%)	1 (3%)	5 (23%)	0 (0%)	3 (33%)	0 (0%)	3 (38%)
	n_c (p_c)	5 (10%)	1 (8%)	3 (8%)	1 (33%)	0 (0%)	5 (23%)	0 (0%)	3 (33%)	0 (0%)	2 (25%)
	n_p (p_p)	1 (2%)	0 (0%)	1 (3%)	0 (0%)	1 (3%)	0 (0%)	0 (0%)	0 (0%)	0 (0%)	1 (13%)
**Thermostable hemolysin**	n_t (p_t)*	51 (98%)	12 (100%)	36 (97%)	3 (100%)	29 (97%)	22 (100%)	17 (100%)	9 (100%)	17 (94%)	8 (100%)
**Nutritional/Metabolic factors**											
**Amonabactin**	n_t (p_t)*	33 (63%)	10 (83%)	21 (57%)	2 (67%)	16 (53%)	17 (77%)	17 (100%)	9 (100%)	0 (0%)	7 (88%)
**Heme uptake system**	n_t (p_t)	52 (100%)	12 (100%)	37 (100%)	3 (100%)	30 (100%)	22 (100%)	17 (100%)	9 (100%)	18 (100%)	8 (100%)
	n_c (p_c)	34 (65%)	10 (83%)	22 (59%)	2 (67%)	17 (57%)	17 (77%)	17 (100%)	9 (100%)	0 (0%)	8 (100%)
	n_p (p_p)	18 (35%)	2 (17%)	15 (41%)	1 (33%)	13 (43%)	5 (23%)	0 (0%)	0 (0%)	18 (100%)	0 (0%)

[a] Flp, fimbrial low-molecular weight protein; MSHA, mannose-sensitive hemagglutinin; RtxA, repeat in toxin A; Tap, type IV *Aeromonas* pilus; T2SS, type II secretion system; T3SS, type III secretion system; T6SS, type VI secretion system. n, number of isolates showing probably complete (c), partial (p), and probably complete or partial (t, total) presence of the specific virulence factor, respectively; p, percentage of isolates showing probably complete (c), partial (p), and probably complete or partial (t, total) presence of the specific

virulence factor, respectively; $_1$, aerolysin A (AerA); $_2$, aerolysin-like hemolysin (ALH); *, All isolates show probably complete presence of the specific virulence factor ($n_t = n_c$, $n_p = 0$; $p_t = p_c$, $p_p = 0\%$). The virulence factors' frequency of occurrence was determined based on Supplementary Table S2 (see sheets "Summary Table" and "Assessment Key"). In general, a virulence factor was considered to be probably completely present if ≥80% of the genes examined in this study were detected. Further details are given in Table 1 and Supplementary Table S2 (sheets "Summary Table" and "Assessment Key").

[b] Including secreted effectors.

[c] Strains were considered aerolysin-positive if aerolysin A (AerA) and/or an aerolysin-like hemolysin (ALH) were present.

[d] C, clinical; G, Germany; cr, coastal region; ncr, non-coastal region; int, intestinal; ext, extraintestinal; *Ac*, *A. caviae*; *Ah*, *A. hydrophila*; *Av*, *A. veronii*; 'Others', The group includes *A. bestiarum* (*n* = 1), *A. dhakensis* (*n* = 1), *A. encheleia* (*n* = 1), *A. salmonicida* (*n* = 2), and *Aeromonas* sp. (*n* = 3). Some percentages are rounded to one decimal place for accuracy.
